# Supplementary material for: Role of healthcare cost accounting in pricing and reimbursement in low-income and middle-income countries: a scoping review
Source: BMJ Open. 2022 Sep 28;12(9):e065019. doi: 10.1136/bmjopen-2022-065019 (PMC10580276; doi:10.1136/bmjopen-2022-065019)
Supplement: Supplementary data [file bmjopen-2022-065019supp001.pdf]

Supplementary materials

Contents

Supplementary materials 1

Structured abstract 2

Table S1. Search strategy 4

Table S2. List of studies identified and included in the review 5

Table S3. Costing evidence for tariff setting in hospital payment schemes in LMICs 7

Structured abstract

Background.

Progress towards universal health coverage requires evidence based policy and price setting informed by good quality cost data systems. Establishing these systems can be complex and both resource and time intensive. and there is little documentary evidence of experiences building such systems in low-and-middle income countries (LMICs).

Objectives.

To synthesise evidence on the experiences of LMICs in institutionalizing cost data systems in order to derive lessons for the technical process of price-setting in the context of UHC goals.

Eligibility criteria.

Studies were included if they identified and/or described either the development of the national tariffs and/or the methods used to estimate or inform the tariffs for hospital services reimbursement.

Sources of evidence,

English-language publications since 2000 indexed in Pubmed, Medline, Econlit and the Web of Science.

Charting methods.

Papers were classified according to whether they explained the technical process of price-setting for reimbursements and whether they reported on the process of primary cost data collection for price-setting. We extracted information on cost data collection methods, outputs and commentary on how cost data was used as well as descriptions of the technical aspects of the tariff setting system and key strengths and challenges. A narrative review approach was used to summarise the evidence by country. Data extraction was performed by one reviewer and then checked by a second reviewer.

Results.

A total of 484 papers were initially identified of which 30 papers were considered eligible. Fourteen papers reported on primary cost data collection for price-setting purposes; 18 papers provided an

explanation of how cost evidence informs tariff-setting. Documented experience is largely focussed in the Asia region (n = 22) with countries at different stages of developing cost systems to inform tariff setting. Country experiences on healthcare cost accounting tend to showcase country costing experiences, methods and implementation. There is little documentation of how cost data has been incorporated into decision making and price setting. Where cost data, cost systems and costing has been used, improved transparency in decision making alongside increased service provision efficiency has followed.

Conclusions.

Countries need to build sustainable cost systems appropriate to their settings and budgets and adopt transparent processes and methodologies for translating costs into prices.

Table S1. Search strategy

Database	Search terms
**Web of Science**	(ALL=(("case*mix" or "cost systems" or "cost*accounting" or "ref*costs" or "resource weights" or "cost*weights" or "national reimbursement" or "DRG" or "hospital payment systems" or "fee*for*service"))) AND ALL=("LMIC" OR "low resource settings" OR "developing countries")
**Econlit**	("case*mix" or "cost systems" or "cost*accounting" or "ref*costs" or "resource weights" or "cost*weights" or "national reimbursement" or "DRG" or "hospital payment systems" or "fee*for*service").mp. [mp=heading words, abstract, title, country as subject]<br><br>No hits when combined with lmics
**Medline**	1. ("case mix" or "DRG" or "case based payment" or "hospital payment system" or "reimbursement" or "resource value unit" or "cost systems" or "cost accounting" or "reference costs" or "resource weights" or "cost weights" or "Price setting" or "service weights")<br>2. "Costs and Cost Analysis"/ or National Health Programs/ or diagnosis-related groups/ or hospitalization/<br>3. 1 OR 2<br>4. Developing Countries/<br>5. "universal health*<br>6. Universal Health Insurance/ or "Delivery of Health Care"/ or Insurance, Health/<br>7. 5 OR 6<br>8. 3 AND 4 AND 7
**PubMed**	("reference cost*" OR "reference price*") AND health* AND national* AND list*)<br>Since 2000

Table S2. List of studies identified and included in the review

Author	Year	Country	Aim of paper in relation to costing (rationale for inclusion)	Type of study	Cost data reported	Comments on country's cost system
Langebrunner JC et al	2009	LMIC (Kazakhstan, Kyrgyzstan)	Manual on methods for setting up provider based systems; provides methods and case study of costing accounting for case based payments	Case studies	Yes	Yes
Joint Learning Network	2014	LMIC	Costing specific resource for lmics	Case studies	Yes	Yes
Martin A	2012	Cambodia	Costing in Cambodian hospital	Primary (cost data)	Yes	Yes
Ministry of Health, Republic of Indonesia	2012	Indonesia	Report on a costing study for Indonesia	Primary (cost data)	Yes	Yes
Ghaffari S et al	2009	Iran	Costing for DRGs in Iran	Primary (cost data)	Yes	Yes
Mathauer I	2011	Kenya	Role of costing in setting insurance reimbursement rates in Kenya	Literature review	Yes	Yes
Jadoo SAA et al	2015	Malaysia	Documenting the development of DRG cost weights in pharmacy in Malaysia	Primary (cost data)	Yes	Yes
Dianingati JK et al	2019	Indonesia	Single site cost data collection to inform price setting	Primary (cost data)	Yes	Yes
Jacobs B et al	2019	Cambodia	Multiple site cost data collection to inform national policy including reimbursement rates	Primary (cost data)	Yes	Yes
Ocharot L et al	2016	Thailand	Cost implications of adverse events in DRG system	Primary (cost data)	Yes	Yes
Prinja S et al	2021	India	Comparing cost data with reimbursement rates in ABPMJAY, India	Primary (cost data)	Yes	Yes
Riewpaiboon, A. et al	2012	Thailand	Development of Relative Value Units for Unit Cost analysis of Medical Services in Thailand	Primary (cost data)	Yes	Yes
Vo TQ et al	2018a	Vietnam	Development of Relative Value Units for Unit Cost analysis of Medical Services in Vietnam	Primary (cost data)	Yes	Yes
Vo TQ et al	2018b	Vietnam	Comparison of hospital costing methods in Vietnam	Primary (cost data)	Yes	Yes
Dianingati RS et al	2021	Asia	Literature review of medical service costs in Asia	Literature review	Yes	No
Jassim AL et al	2011	India	Testing for RVU method for costing in hospitals in India	Primary (cost data)	Yes	No
Chatterjee S et al	2013	India	Hospital costing study	Primary (cost data)	Yes	No
Stenberg K et al	2018	Global	Estimating unit costs of health services at a country level based on global dataset	Primary (cost data)	Yes	-
Lian LL et al	2014	Taiwan	Assessing incentives in DRGs		No	Yes
Barber S et al	2019	Global (India, Malaysia, Thailand)	Manual and case studies on price setting for case-based payment	Case studies	No	Yes
Bredenkamp C et al	2020	Global (China, Kyrgyz Republic, Thailand)	Case studies of DRG transitions	Case studies	No	Yes

Author	Year	Country	Aim of paper in relation to costing (rationale for inclusion)	Type of study	Cost data reported	Comments on country's cost system
Zhao C et al	2018	China	Document China's experiences with shifting to case based payment schemes	Case studies	No	Yes
Prinja S et al	2020	India	Commentary on cost data for policy	Commentary	No	Yes
Mathauer I et al	2013	LMIC	Literature review of DRG experiences in LMICs	Literature review	No	Yes
Doshmangir L et al	2020	Iran	To document Iran's experience of tariff setting	Primary	No	Yes
Hoang VM, et al	2014	Vietnam	Reporting on costing in Vietnam for provider payment reform	Primary	No	Yes
National Health Authority	2019	India	Describes process of updating HBP package rates	Primary	No	Yes
Patcharanarumol K et al	2018	Thailand	Comparing strategic purchasing in two financing schemes	Primary	No	Yes
Rasiah, D et al	2011	Malaysia	Comparing methods for costing of health services in Malaysia	Primary	No	Yes
KPMG	2019	India	Overview of AB-PMJAY reform and financing mechanisms	Report	No	Yes
Barber S et al	2020	Global	To provide policy recommendations on estimating the cost of UHC	Case studies	No	Yes
Wagstaff A et al	2007	East Asia	Lessons learned in Asia for financing reforms including provider payment	Primary	No	Yes
Hu S et al	2008	China	Commentary	Commentary	No	No
Zeng W et al	2018	Global	Examining role of PBF in strengthening health systems	Commentary	No	No
Beck E et al	2012	LMIC	Describes the financial information required by policy makers and other stakeholders to enable them to make evidence-informed decisions and reviews the quantity and quality of the financial information available,	Literature review	No	No
Zou K et al	2020	China	Literature review of impact of case based payments in China	Literature review	No	No
Bertram M et al	2017	Global	Estimating unit costs for disease control programmes	Primary	No	No
Immunisation Costing Action Network	2018	LMIC	Costing of immunisation programmes	Primary	No	No
Jian W et al	2016	China	Assessing capacity of information system to implement DRGs (not cost system)	Primary	No	No
Watkins D et al	2020	LMIC	Resource requirement estimation of model health benefit packages (essential services)	Primary	No	No

Table S3. Costing evidence for tariff setting in hospital payment schemes in LMICs

Year	Author	Country/ Region	Type of study	Method of cost data collection	Generated output (what form does the cost data take e.g. Unit costs, DRG cost weights, base rates)	What is/was the cost data used for or what is the intended use
2021	Prinja S et al	India	Primary (cost data)	Step down allocation; mix of top down and bottom-up costing	Reference costs to inform price setting and HTA	Comparison of health benefit package reimbursement rates with costs; use in HTA
2021	Dianingati RS et al	Asia	Literature review	Methods used in hospital cost analysis in Asia include direct allocation, step-down allocation, simultaneous equation allocations, micro costing and simplified activity-based costing.	Different final outputs include average costs, patient level (micro) costs, RVUs and RCCs.	Not reported for the studies in the review
2019	Jacobs B et al	Cambodia	Primary (cost data)	Step down allocation; bottom-up costing	Costs of health services	Initial phase of establishing a routine costing system for health services
2019	Dianingati JK et al	Indonesia	Primary (cost data)	Step down allocation: micro costing	Unit cost of the laboratory services of a district hospital in Indonesia	To compare actual costs of laboratory services with government established reference prices
2018a	Vo TQ et al	Vietnam	Primary (cost data)	Step down allocation; Micro-costing	Relative value units (RVUs) for hospital services	Develop a set of RVUs for hospital services in Vietnam
2018b	Vo TQ et al	Vietnam	Primary (cost data)	1. Step down allocation; Micro-costing. 2. Hospital charges	Relative value units (RVUs) for hospital services	Comparison of different methods and identification of best method for the estimation of RVUs
2018	Stenberg K et al	Global	Primary	WHO-CHOICE service delivery unit costs based on regression analysis of country data sets	Cost per bed-day and cost per outpatient visit	Address cost information gap by producing standardised cost estimates that are comparable across countries
2016	Ocharot L et al	Thailand	Primary (cost data)	Hospital charges	Uncompensated adverse event costs for hospitalised patients	To demonstrate the cost implications of patient safety
2015	Jadoo SAA et al	Malaysia	Primary (cost data)	Step down allocation; bottom-up costing	DRG cost weights for pharmacy services	To identify the actual cost of pharmacy services by DRG case-mix group and inform reimbursement rates
2014	JLN	India Aarogyasri Hospital	Case study	Top down, direct and indirect costing for operating and capital costs.<br>For the cost of benefits packages a bottom-up approach was used. (4 hospitals; 42 procedures)	Costs of benefit packages	To set rates for 938 new benefit packages and revise estimates for pre-existing packages

Year	Author	Country/ Region	Type of study	Method of cost data collection	Generated output (what form does the cost data take e.g. Unit costs, DRG cost weights, base rates)	What is/was the cost data used for or what is the intended use
2014	JLN	Indonesia casemix	Case study	Top-down costing (137 hospitals)	Not specified	To estimate the cost of health services and construct cost weights for case based payments.
2014	JLN	Indonesia Health Facility	Case study	Top-down approach for both recurrent costs and capital costs. Bottom up approach to cost specific episodes of illness (200 hospitals)	Unit costs and episodes of illness	To estimate the production cost of services and drivers of cost variation among providers<br>Capitation payments
2014	JLN	Central Asian Republic	Case study	Predominantly top down, but also bottom up to obtain allocation statistics (15 hospitals)	Cost per bed-day by department	To estimate the cost per bed-day by cost centres to inform DRG weight coefficients<br><br>There was sufficient data available for top down costing which could be conducted in a relatively short period of time
2014	JLN	Malaysian DRG	Case study	Top-down approach to measure and value personnel, drugs/medical supplies, overheads, and capital resource use. Bottom up planned for ICU stays, laboratory tests and radiological interventions. (10 hospitals)		To determine the cost of delivering health services in government hospitals to inform budgetary requirements<br><br>Top down approach was used most of the time, but for items that were heterogenous in their resource use or expensive a bottom up approach was more suitable
2014	JLN	India (PHFI)	Case study	Mixed method approach. Top down for the cost of resources consumed. Bottom up for personnel. (5 hospitals)	JLN costing exercise	PHFI used a mixed-method approach because data on resource use were not always available at the department level
2014	JLN	Phillipines	Case study	Analysis of claims. All tertiary hospitals.		Cost of health services and specific disease categories
2013	Chatterjee S et al	India	Primary (cost data)	Step down allocation; bottom up costing	Cost per OP visit, cost per bed day; cost per emergency visit, cost per OT case	Informs hospital administrators, to improve efficiency and demonstrate the feasibility of hospital cost analysis in India

Year	Author	Country/ Region	Type of study	Method of cost data collection	Generated output (what form does the cost data take e.g. Unit costs, DRG cost weights, base rates)	What is/was the cost data used for or what is the intended use
2012	Riewpaiboon A.	Thailand	Primary (cost data)	1. Reimbursement price list; 2. Bottom up/step down allocation; 3. Patient interviews	1. RVUs; 2. inpatient base rate; 3. patient non-medical costs	To develop a list of standard unit costs of medical services for Thailand for HTA.
2012	Ministry of Health, Republic of Indonesia	Indonesia	Primary (cost data)	Step-down accounting methodology	For hospital services: cost per outpatient, cost per inpatient and cost per bed-day	To better understand the cost of delivering health services across the country and inform policy on geographic resource allocation, the development of hospital payment system and capitation formulae.
2012	Martin A	Cambodia	Primary (cost data)	Top down costing/step-down cost accounting (10 hospitals)	Average cost per discharge, per inpatient data and per outpatient visit by hospital department	To link costs with funding sources and document use of funds as well as inform the revision of the National Charter on Health Financing including provider payment reforms.
2011	Mathauer I	Kenya	Literature review	Most recent, multi-site costing studies<br>1. Bottom up, ingredients costing from (11 faith-based provider hospitals -); 2. Step down cost accounting model (22 private for profit hospitals)	Cost per case (surgical, non-surgical and outpatients); cost per bed-day (surgical and non-surgical)	Costing information to inform resetting health insurance remuneration rates
2011	Jassim AL et al	India	Primary (cost data)	Step down allocation; process costing	Cost per relative value unit in support cost centres (e.g. laboratory)	To improve costing accuracy and provide guidelines for improved costing at the hospital level
2009	Ghaffari S et al	Iran	Primary (cost data); cost modelling	Primary: Step down allocation; ABC costing<br>Cost modelling: DRG cost weights imported from Australia	Relative value units, DRG costs and cost weights using indirect costs, direct care services and costs of care	To guide costing efforts for case mix funding models and identify optimal method for costing given data constraints.
2009	Langebrunner JC et al	Kyrgyzstan	Case studies	Cost accounting: Step down cost allocation to departmental level, Adapted from US medicare cost reports (initially 1 public hospital)	Cost per bed day and cost per case	Informed case base payment rates in DRG system
