# Supplementary material for: Role of healthcare cost accounting in pricing and reimbursement in low-income and middle-income countries: a scoping review
Source: BMJ Open. 2022 Sep 28;12(9):e065019. doi: 10.1136/bmjopen-2022-065019 (PMC10580276; doi:10.1136/bmjopen-2022-065019)
Supplement: Author's manuscript [file bmjopen-2022-065019.draft_revisions.pdf]

BMJ Open is committed to open peer review. As part of this commitment we make the peer review history of every article we publish publicly available.

When an article is published we post the peer reviewers' comments and the authors' responses online. We also post the versions of the paper that were used during peer review. These are the versions that the peer review comments apply to.

The versions of the paper that follow are the versions that were submitted during the peer review process. They are not the versions of record or the final published versions. They should not be cited or distributed as the published version of this manuscript.

BMJ Open is an open access journal and the full, final, typeset and author-corrected version of record of the manuscript is available on our site with no access controls, subscription charges or pay-per-view fees (http://bmjopen.bmj.com).

If you have any questions on BMJ Open's open peer review process please email info.bmjopen@bmj.com

BMJ Open

The role of healthcare cost accounting in pricing and reimbursement in low- and middle-income countries

Journal:	*BMJ Open*
Manuscript ID	bmjopen-2022-065019
Article Type:	Original research
Date Submitted by the Author:	25-May-2022

Complete List of Authors:	GUINNESS, LORNA; Center for Global Development<br>Ghosh, Srobana; Center for Global Development<br>Mehndiratta, Abha; Center for Global Development<br>Shah, Hiral A.; Imperial College London, Department of Infectious Disease Epidemiology; Center for Global Development

Keywords:	Health economics < HEALTH SERVICES ADMINISTRATION & MANAGEMENT, Health policy < HEALTH SERVICES ADMINISTRATION & MANAGEMENT, International health services < HEALTH SERVICES ADMINISTRATION & MANAGEMENT

SCHOLARONE™
Manuscripts

I, the Submitting Author has the right to grant and does grant on behalf of all authors of the Work (as defined in the below author licence), an exclusive licence and/or a non-exclusive licence for contributions from authors who are: i) UK Crown employees; ii) where BMJ has agreed a CC-BY licence shall apply, and/or iii) in accordance with the terms applicable for US Federal Government officers or employees acting as part of their official duties; on a worldwide, perpetual, irrevocable, royalty-free basis to BMJ Publishing Group Ltd ("BMJ") its licensees and where the relevant Journal is co-owned by BMJ to the co-owners of the Journal, to publish the Work in this journal and any other BMJ products and to exploit all rights, as set out in our licence.

The Submitting Author accepts and understands that any supply made under these terms is made by BMJ to the Submitting Author unless you are acting as an employee on behalf of your employer or a postgraduate student of an affiliated institution which is paying any applicable article publishing charge ("APC") for Open Access articles. Where the Submitting Author wishes to make the Work available on an Open Access basis (and intends to pay the relevant APC), the terms of reuse of such Open Access shall be governed by a Creative Commons licence – details of these licences and which Creative Commons licence will apply to this Work are set out in our licence referred to above.

Other than as permitted in any relevant BMJ Author's Self Archiving Policies, I confirm this Work has not been accepted for publication elsewhere, is not being considered for publication elsewhere and does not duplicate material already published. I confirm all authors consent to publication of this Work and authorise the granting of this licence.

For peer review only

The role of healthcare cost accounting in pricing and reimbursement in low- and middle-income countries: a scoping literature review

Lorna Guinness (ORCID -0000-0002-1013-4200), PhD, Center for Global Development, London, United Kingdom

Srobana Ghosh, MSc, Center for Global Development, London, United Kingdom

Abha Mehndiratta, MBBS, DCH, DNB, MPH, Center for Global Development, London, United Kingdom

Hiral Anil Shah (ORCID - 0000-0003-0204-451X), MRC Center for Global Infectious Disease Analysis, Department of Infectious Disease Epidemiology, School of Public Health, Imperial College London, London, UK.

Corresponding author

Lorna Guinness
Great Peter House,
Abbey Gardens,
Great College St,
London
SW1P 3SE
Email: lornaguinness@gmail.com
Phone: +447926674052

Word count:

3959

For peer review only - http://bmjopen.bmj.com/site/about/guidelines.xhtml

Abstract

Objectives: Progress towards universal health coverage (UHC) requires evidence based policy and good quality cost data systems. Establishing these systems, can be complex, resource intensive and take time. This study synthesises available evidence on the experiences of low-and-middle income countries (LMICs) in trying institutionalize cost data systems in order to derive lessons for the technical process of price-setting in the context of UHC goals.

Design: A scoping literature review, screening for publicly available peer-reviewed English-language publications alongside a grey literature search, and narrative synthesis approach

Setting: National level health systems in low- and middle-income countries

Interventions: The use of cost evidence in price-setting for case-based payments

Results: A total of 484 papers were initially identified of which 30 papers were considered eligible. Fourteen papers reported on primary cost data collection for price-setting purposes; 18 papers provided an explanation of how cost evidence informs tariff-setting. Documented experience is largely focussed in the Asia region (n = 22) with countries at different stages of developing cost systems to inform tariff setting. Country experiences on healthcare cost accounting tend to showcase country costing experiences, methods and implementation. There is little documentation of how cost data has been incorporated into decision making and price setting. Where cost data, cost systems and costing has been used, improved transparency in decision making alongside increased service provision efficiency has followed.

Conclusions

While there are accepted and widely used methods for generating cost information, countries need to build sustainable cost systems appropriate to their settings and budgets and adopt transparent processes and methodologies for translating costs into prices.

For peer review only

STRENGTHS AND LIMITATIONS OF THIS STUDY

- Although the process of payment reform has been well documented, the scoping review found that how cost and price evidence is used in the price-setting process in LMICs is poorly documented
- The review relied on publicly available information and there maybe further information that was not accessible.
- The terminology on the role of cost evidence in price-setting in the literature is poorly defined and inconsistent.
- The review explored both costing methods used in cost systems and how cost information is used to inform price setting
- The review found consistent themes around the need to use cost information using a systematic methodology, reporting this transparently and working with providers to develop the system.

For peer review only

For peer review only - http://bmjopen.bmj.com/site/about/guidelines.xhtml

INTRODUCTION

Low-and-middle income countries (LMICs) have been making significant progress towards universal health coverage through innovative healthcare financing. One focus of healthcare financing reforms has been reimbursement schemes that target the explicit goals of efficiency and cost containment while improving quality and reaching the poor and vulnerable. Historically, block grants have been used to reimburse healthcare providers in publicly financed systems in LMICs. However, as national-level public purchasers have evolved and a broader range of healthcare providers (e.g. private or faith-based healthcare providers) are accepted as part of the developing health system, newer prospective payment mechanisms and systems of provider reimbursement are being used by government purchasers of healthcare [1].

Common prospective payment mechanisms such as case-based payments for the reimbursement of secondary and/or tertiary care and capitation payments for primary care providers are now being championed across developing regions and countries. Case-based payments are equivalent to a system where providers are reimbursed based on cases treated rather than per service or per bed days [2]. On the other hand, capitation based payments are equivalent to a payment system where lump-sum payments are made to care providers based on the number of patients in a target population [2,3].

Setting reimbursement rates requires a reliable cost evidence base to enable price negotiations that are transparent, facilitate cost control and help drive providers to more efficient services. In principle, information is needed on the average cost per case across all admissions and/or visits (a base rate) and the relative value of different conditions as classified in the respective country (e.g., Diagnosis Related Groups, specialty-based classification, intervention specific health benefit package etc) [4–6]. In a case-based payment scheme, the service groups are often DRGs or a similar grouping system which provides a means of relating the type of patients a hospital treats to the costs incurred by the hospital. For capitation-based systems the grouping is related to the average expected cost of treating a patient under the care of the provider. In both types of system, the technical process of price-setting requires a robust cost system to be in place, using principles that can be guided or even mandated by a purchaser, in order to generate reliable health service cost estimates.

For peer review only - http://bmjopen.bmj.com/site/about/guidelines.xhtml

Raulinajtys-Grzybek (2014) defines the cost system as "*a cost accounting system that ensure the cost homogeneity of individual groups* (of services)" [7]. There are however variation in costing systems across health systems as a result of choices about the process of collecting and verifying the data, the stage of development of the reimbursement system, the regulation around cost accounting and the costing methodology used [7]. For example, they can vary from one off costing studies to regular national costing surveillance [7,8]. Some cost surveys involve all participating providers e.g., in the UK and Australia all providers are mandated to submit cost accounting information; in others, only a sample of representative providers is used e.g. France, Germany and Thailand [9].

In terms of costing methodology, according to Gapenski and Reiter (2016) "the holy grail of cost estimation is costing at the service or individual patient level" [10]. More advanced systems e.g., those in the UK and Australia use bottom-up style costing methods to derive patient level DRG costs [11]; but there are simplified methods available that calculate the average cost of procedure through step down allocation methods [12]. Whichever approach is taken, it is important that the costing is nationally acceptable and can capture structural differences in cost that might be present (types of provider, demography, geography etc) as well as variability between the cost of the conditions treated. In addition, the national costing system should be standardised across providers, creating transparency and comparability [8].

In LMICs, while the process of payment reform has been well documented, there is less information available about the role of cost information in the technical process of setting reimbursement rates. Increasing number of countries are moving towards case-based payment schemes for secondary care within their UHC strategies. Documenting the cost systems used to generate evidence for rate setting can provide lessons for the further development of existing systems or the establishment of new ones. The aim of this paper is therefore to synthesise the evidence on the role of cost accounting in setting reimbursement rates for case-based payment schemes in LMICs. We perform a scoping literature review and narrative synthesis to document the current practice in LMICs based on publicly available information and recommend steps for the technical process of price-setting in LMICS in the context of UHC goals.

For peer review only - http://bmjopen.bmj.com/site/about/guidelines.xhtml

METHODS

Search Strategy and Selection Process

A scoping review approach was used to synthesise the evidence on cost accounting in LMICs. We aimed to map the body of literature, clarify key concepts and identify any gaps in the research [13]. We further refined our research question using a standard PICO framework:

- Problem: technical process for price setting for hospital case-based payments in LMICs
- Intervention: cost systems
- Comparator: non-cost-based methods
- Outcome: improved evidence base for decision-making

We used several approaches in identifying the literature. First, we conducted a search of the literature for peer-reviewed English-language publications indexed in Pubmed, Medline, Econlit and in the Web of Science on the subject of national level health system costing in LMICs and the associated design of their costing systems. Our search was conducted using the following terms: ("case*mix" or "cost systems" or "cost*accounting" or "ref*costs" or "resource weights" or "cost*weights" or "national reimbursement" or "DRG" or "hospital payment systems" or "fee*for*service") AND ("LMIC" or "low resource settings" or "developing countries"). We conducted a search that included the country name of all LMICs, as defined by the World Bank. To complement this, we consulted existing libraries of both grey and peer-reviewed literature held by the research team. We then conducted an analysis of text words contained in the title and abstract to help identify further keywords and index terms. A further search was then conducted using the identified keywords and index terms. Finally, the reference list of all identified reports and articles were reviewed for any reports or papers that might have been missed. The search strategy is provided in the Supplement (Table S1).

Eligibility Criteria & Screening

Papers in the English language and published since 2000 were included. Results were then hand screened to ensure that the topic was limited to the eligible countries (LMICs as defined by the World

For peer review only - http://bmjopen.bmj.com/site/about/guidelines.xhtml

Bank) and that the study identified and/or described the development of the national tariffs for hospital reimbursements and/or the methods used to estimate or inform the tariffs for hospital services reimbursement. The titles and abstracts were screened independently by two reviewers as per the inclusion and exclusion criteria defined by the study. The second screen involved reviewing full texts.

Data Extraction & Synthesis

The papers were then classified according to whether they explained the technical process of price-setting for reimbursements (i.e., if and how cost data was used) and whether they reported on the process of primary cost data collection for price-setting. For those papers or case studies reporting on the process of primary cost data collection for price setting, we extracted information on the method of cost data collection, the output and any commentary on how the cost data was used for price setting for hospital case-based payments including identifying the commissioning agency. From the papers that described how cost data is used in price setting, we extracted information on any description of the technical aspects of the tariff setting system in place, at the time of the study, and the key strengths and challenges of the approach used. For those papers describing more than one country experience, only evidence on LMIC experience was extracted. We use a narrative review approach to summarise the evidence by country. Data extraction was performed by one reviewer and then checked independently.

Patient and Public Involvement

Neither patients of public were involved in the design, conduct, reporting or dissemination plans of our research.

RESULTS

Overview of the literature

A total of 484 papers were initially identified of which 424 papers were excluded in the initial screening. The second screen involved reviewing full texts, leading to the inclusion of 30 papers in the review as described in the PRISMA diagram in Figure 1.

For peer review only - http://bmjopen.bmj.com/site/about/guidelines.xhtml

For peer review only

Of the 30 papers extracted (see Supplement Table S2), 7 papers stated a global focus (including LMICs) [1,4,14–18] and one paper reported to be focussed on the Asia region [19] (see Figure 2). Of the single country focussed papers, 6 related to India [20–25]. We found 3 studies each related to Thailand [6,26,27] and Vietnam [28–30]. There were 2 studies focussed on each of Indonesia [31,32], Iran [33,34], Malaysia [35,36] and Cambodia [37,38] and one study each for Kenya [39] and China [40]. Further, within the global papers, we identified case studies on: Kazakhstan, Kyrgyzstan, India, Malaysia, Thailand, and China.

Papers reporting on primary collection of cost data to inform tariff-setting

Twenty-three case studies from 14 fourteen studies reported on primary cost data collection for price setting purposes in a single country setting, either describing methods or both methods and results (see Supplement Table S3). Twelve case studies also had the explicit aim of generating cost information for broader policy processes. In terms of pricing, two case studies reported on a costing exercise that was designed to inform capitation payment rates [16,31], six studies aimed at generating cost weights for DRGs [4,16,36][1] or unspecified case groups [16,27,29][2] and three papers reported on estimation of the cost of health benefit packages [16,25][3]. A final case study reviewed the available cost evidence for informing price setting in the National Health Insurance Fund, Kenya [39].

For the studies reporting costs, cost per service unit at the hospital level was the most frequently sited output e.g., cost per bed day, cost per admission and cost per outpatient visit. Three studies generated unit costs for specific services: cost per adverse event [26]; laboratory services [32]; and pharmacy services [36]. And a further three studies generated costs of health benefit packages [16,25][3]. Relative value units were the primary output of 7 studies, one of which also explicitly estimated an inpatient base rate [27].

Fourteen of the case studies were commissioned by the local ministry of health or agency acting on their behalf. However, in many cases, it was not clear who had commissioned the costing or if the

[1] Joint Learning Network case studies: Central Asian Republics
[2] Joint Learning Network case studies: Indonesia Ministry of Health
[3] Joint Learning Network case studies: PhilHealth and India Aaorgyasri

For peer review only - http://bmjopen.bmj.com/site/about/guidelines.xhtml

study was linked to the national policy process [14,16,23,24,26,29,30,36][4]. Two studies evaluated different methods for generating robust relative value units [30,34].

Papers reporting on how cost data informs the tariff-setting process

We identified 18 papers that provided explanation of the technical process of tariff-setting documenting experiences in 10 different countries (see Figure 3). Eight papers were published since the beginning of 2020, 8 papers in the period 2015-2019, 4 papers in the period 2010-2014, and 2 papers were published before 2010 (see Figure 4). The papers provided mixed levels of detail on the technical processes of price-setting and the strengths and weaknesses in each locality. The current tariff system, presence of an explanation of the price-setting process, the data used in price setting and resulting policy levers and implications are summarised in Figure 5.

Only one study described tariff setting in Africa [39]. The paper reviewed the available evidence on costs for informing Kenya's National Health Insurance Fund prices and was published in 2011. Although the cost information were considered reliable by all stakeholders, in part due to their involvement in the costing exercises, the costs had not been used for setting prices at the time of the study.

The other countries covered were all in Asia. In the Central Asia region, three papers focus on the reform of the tariff setting system in Kyrgyzstan [4,15,18]. The technical process of price setting is clearly documented. This process includes cost control measures derived from linking the reimbursement rates to the MOH budget. The Thai Universal Coverage Scheme (UCS) also provides an example in which cost control is built into the base rate through linkages to the budget. However, as figure 5 states, in Thailand, there are 3 government funded and implemented schemes. Although all Thai schemes use the same Thai-DRG grouper, the Civil Service Medical Benefit Scheme (CSMBS) and Social Health Insurance (SHI) schemes do not use cost control mechanisms, as the rates are not linked to an overall budget and are different rates for different hospitals [1,6,18,27].

[4] Joint Learning Network case studies: India – Public Health Foundation of India

For peer review only - http://bmjopen.bmj.com/site/about/guidelines.xhtml

The Kyrgyz reforms were found to be vulnerable to gaming as the system does not make full use of the data available potentially leading to misclassification of diagnoses. This potential for gaming is also highlighted in Iran. In contrast to the systematic introduction and use of cost accounting in Kyrgyzstan, Iran's price setting process involves a technical assessment by an independent body but with limited transparency (see Figure 5) [33,34]. Doshmangir et al note that without an objective and explicit mechanism in the updating of medical tariff and no structure to effectively manage conflicts of interest, the pricing system has in effect become "a tool for revenue manipulation" [33].

Challenges also arise when reimbursement rates are not based on cost evidence. In India's national insurance programme for the poor and vulnerable, the government used existing information to set reimbursement rates while establishing a review system to allow for modification and improvement over time (see Figure 5) [15]. However, the method in which information from costing studies, experts and rates under previous schemes is compiled is not transparently reported on. Studies show that the rates vary considerably from actual costs (42% of HBPs had a price less than 50% of the true cost in 2018) [25] . This could affect the recruitment of providers, the coverage and quality of care, and bring the rates themselves into doubt [41]. Indonesia faces a similar problem in respect of laboratory services. Dianingati et al, report on a lack of transparency in the development of the reference prices set by the government and that the true costs of service delivery are 40-53% higher than the reference price. However, validation of the rates is difficult as data on the cost of healthcare services is still limited to a few services, in focal geographical areas, restricted to the public sector with few published and readily accessible cost data analyses/ data sets [20,25].

Both the Thai UCS and Kyrgyz price-setting systems use cost accounting to inform their base rates and case weights. Langebrunner et al note how cost accounting has been used as an evaluation tool and allowed for tariff adjustments based on evidence so that payments match services in Kyrgyzstan. Similarly, in the Thai UCS scheme, a key feature of the tariff setting is the cost information on which pricing for the UCS is based. This is collected on a periodic basis in a cost survey and has evolved from initial work using an RVU method and "top level" hospital cost data [27] to a 900 hospital

For peer review only - http://bmjopen.bmj.com/site/about/guidelines.xhtml

survey. While no study described how the system has reformed, the papers note that the gradual, step wise implementation allowed for institutional and technical capacity building.

There was less detail reported on the tariff setting process in China, Indonesia, Malaysia and Vietnam. The one study documenting tariff setting processes in China raises concerns that the schemes used retrospective payment system and fails to build in efficiency and cost control [18]. In Vietnam there was also concern that the fees and the payment schemes bore little relationship to the costs of delivering services although the relative value unit method used to calculate rates for the capitation scheme was relatively simple. Similarly, while the Malaysian system was designed for global budgeting, it also demonstrates that pricing evidence can be based on skeletal data sets such as those that focus on large expenditure items and patient data that are feasible to collect [1].

DISCUSSION

Cost systems increase the policy evidence base

Cost systems can create a transparent evidenced based process for price-setting and these systems need to generate good quality data, based on accepted methodologies. Studies from Iran and Thailand emphasise how important the cost system is in the setting of health benefit package/ DRG prices, to minimise gaming and prevent cost escalation [6,33]. Leaving base rates open to negotiation at the individual provider level with minimal evidence on costs and efficiency of service provision, leaves the system vulnerable to gaming.

Cost evidence can increase efficiency of service provision

Creating a tariff setting system that does not use costs based on empirical evidence can embed inefficiencies and possibly make it more difficult to implement costing in the future [33]. A centralised cost accounting system, such as was developed in Kyrgyzstan, was considered a major strength of the broader health system reforms – allowing for policy reform to anticipate expenditure needs and enabling the government to effect change more effectively.

For peer review only - http://bmjopen.bmj.com/site/about/guidelines.xhtml

Some data is better than no data but its important to plan early for sustainable institutionalized *cost systems*

On the other hand, it is also recognised that such exercises are expensive, and a persistent issue is that of overcoming the capacity constraints to generate this information. While obtaining good quality cost information can be an important aid to evidence-based decision-making, it is important to be aware of the trade off in accuracy and resources needed to generate the information. Where resources are highly constrained, any data can be better than no data, particularly if the data are reported transparently and how the data informs decisions is clearly communicated and accounted for. If cost accounting is not the norm and the budget is limited, costing for price setting may need to start with simpler methods, using, for example, expenditure data, relative value units and smaller samples of facilities. Alternative approaches, for example in India, Cambodia and Kenya, have started with the implementation of baseline multi-site costing studies. Although these are one off exercises, and therefore cannot identify drivers of efficiency, they provide an evidence base and good practice on which to build. The costing itself can be a way to bring stakeholders into the price setting process and build capacity for future costings.

The example of Kyrgyzstan shows how implementing a cost system is a slow, gradual and complex process. The established costing systems identified in the literature illustrate how a cost system has evolved from one-off exercises and developed into complex system with increasing numbers of participating providers (Thailand, Kyrgyzstan, China).

Costing needs and methods vary and need to be appropriate for the setting

Methods for cost data collection also need to be appropriate for the setting. Studies from Thailand and Vietnam compare different approaches to obtaining the base rate and cost weights for health technology assessment and pricing. They compare micro costing with relative value unit approaches and find both to be feasible with micro-costing being highly resource intensive. The costing methods tend to follow the same principles using top-down allocation methods supplemented with bottom-up costing if resources allow, for some specific inputs. In Malaysia, one study demonstrated the

For peer review only - http://bmjopen.bmj.com/site/about/guidelines.xhtml

feasibility of using the electronic prescribing system to generate DRG weights, although it was recognised that these were not available in most facilities.

A transparent set of principles for translating cost evidence into base rates and weights is needed

As well as cost data collection, a systematic method for translating costs into prices or reimbursement rates helps avoid skewed incentives within the prices, evident in the unexplained differences between costs and reimbursement rates found in India and Indonesia [25,32]. Langenbrunner's reporting of the Kyrgyzstan case study provides the most comprehensive description for the calculation of the base rate and case-based weightings and how to use these to set reimbursement rates [4]. Patharanarumol et al also describe the principles applied for estimating the base rates and weights in the Thai UCS scheme [6]. For both settings, explicitly accounting for the budget in the price estimation using an "economic adjustment" is a key mechanism of cost control. This level of transparency is not apparent elsewhere in the literature identified. For example, while the different strands of information used for price setting are documented in the reports on India, the method for combining this information is not reported [25,42]. And, in Iran, the lack of such a methodology was reported as a significant problem for the DRG system as a whole leading to price manipulation by different stakeholders [33].

Limitations

The scoping review has found a very limited level of evidence around how cost and price evidence is used in the price-setting process at the country level. In addition, many of the studies are old and may be outdated. While this can be in part due to the infancy of many of the price-setting systems and reforms, the nature of the review may have limited the evidence generated. While early reforms might be reported for some countries, it was not possible to determine how the tariff-setting processes have evolved and are currently being implemented. For example, Vietnam's pilot study was published in 2014 but there were no corresponding papers documenting next steps; nor did we identify more recent reports on Kenya and Cambodia where costing evidence from large multi-site studies were identified. Further, the terminology required to search for the role of cost evidence in price-setting in the literature is poorly defined. The terms cost and price are used in many different ways to mean

For peer review only - http://bmjopen.bmj.com/site/about/guidelines.xhtml

different things which may have led to some omissions. In addition, during the screening process many articles were identified on the process of developing DRG type reforms, but few focussed on the price-setting process and how cost evidence is used in the price-setting process. To address this we extended the search and performed additional searches using the key words identified in the initial papers found that met the inclusion criteria. Our review of the grey literature was limited to a google search and snowballing from references that were identified in the initial search. It is likely evidence in this area lies in government and donor reports and we may have missed these. Restricting reports to the English language may have compounded this.

Despite these limitations and the few country case studies identified, consistent themes were evident around the need to use cost information using a systematic methodology, reporting this transparently and working with providers to develop the system. Our review confirms that cost evidence can increase efficiency of service provision by increasing the policy evidence base. To generate this evidence, countries need to build cost systems appropriate to the setting and data availability but allowing for and investing in increasing complexity as data systems improve. Even while national costing surveillance should be an aspiration, prices should be set using cost evidence which can take the form of one off costing studies, or even hospital charges. Especially in the absence of national surveillance, the method in which these data are then used to set base rates and price weights should be part of a transparent process that involves all stakeholders and takes account of heterogeneity in costs driven by demand side (e.g. condition or patient specific) and supply side (e.g. hospital location) factors.

CONCLUSION

LMICs are increasingly turning to insurance-based models of healthcare and private sector providers to increase coverage of the poor and vulnerable. To help achieve value for money within these universal health coverage goals, publicly financed insurance schemes need to account for budget constraints, encourage efficient health service delivery and use good quality evidence transparently in setting reimbursement rates. Documentation of the good practice and the challenges of generating cost evidence and creating costing systems for informing reimbursement decisions in resource poor

For peer review only - http://bmjopen.bmj.com/site/about/guidelines.xhtml

settings are lacking. While there are accepted and widely used methods for generating cost information, countries need to build more sustainable cost systems and adopt more transparent systems and methodologies for translating costs into prices.

DECLARATIONS

Ethics approval and consent to participate:

Not applicable

Consent for publication:

Not applicable

Availability of data and materials:

The datasets used and analysed during the current study are available from the corresponding author on reasonable request.

Competing Interests

HS contributed to this study whilst being employed for the Center for Global Development. HS is now an employee for GSK and holds shares in the GSK group of companies; all other authors declare no competing interests. LG declares fees for postgraduate teaching at London School of Hygiene and Tropical Medicine.

Funding

This work was supported by the Bill & Melinda Gates Foundation Grant no: INV-003239.

Authors' Contributions

- Concept and design: LG, HAS, SG, AM
- Acquisition of data: LG, SG
- Analysis and interpretation of data: LG, SG
- Drafting of the manuscript: HAS, LG, SG
- Critical revision of paper for important intellectual content: HAS, LG, SG

For peer review only - http://bmjopen.bmj.com/site/about/guidelines.xhtml

- Obtaining funding: LG, AM
- Supervision: LG, AM

Acknowledgements

Grateful thanks to Yi-Ling Chi and Lydia Regan at Center for Global Development for valuable comments and advice to the search strategy.

REFERENCES

1 Barber SL, Lorenzoni L, Ong P. *Price Setting and Price Regulation in Health Care*. OECD 2019. doi:10.1787/ed3c16ff-en

2 Tan SY, Melendez-Torres GJ. Do prospective payment systems (PPSs) lead to desirable providers' incentives and patients' outcomes? A systematic review of evidence from developing countries. *Health Policy and Planning* 2018;**33**:137–53. doi:10.1093/HEAPOL/CZX151

3 Robyn PJ, Sauerborn R, Bärnighausen T. Provider payment in community-based health insurance schemes in developing countries: a systematic review. *Health Policy and Planning* 2013;**28**:111–22. doi:10.1093/heapol/czs034

4 Langenbrunner JC, Cashin C, O'Dougherty S. *Designing and Implementing Health Care Provider Payment Systems : How-To Manuals*. 2009.

5 Government of Australia. Australian Public Hospitals Cost report. 2016.https://www.ihpa.gov.au/publications/australian-public-hospitals-cost-report-2013-2014-round-18

6 Patcharanarumol, W Panichkriangkrai, W Sommanuttaweechai, A Hanson K, Wanwong, Y Tangcharoensathien V. Strategic purchasing and health system efficiency: A comparison of two financing schemes in Thailand. *PLoS ONE* 2018;**13**.https://pubmed.ncbi.nlm.nih.gov/29608610/ (accessed 20 Jan 2021).

7 Raulinajtys-Grzybek M. Cost accounting models used for price-setting of health services: An international review. *Health Policy* 2014;**118**:341–53. doi:10.1016/j.healthpol.2014.07.007

8 Bredenkamp C, Bales S, Kahur K. Transition to Diagnosis-Related Group (DRG) Payments for Health Lessons from Case Studies. Washington DC: 2020.

9 Barber SL, Lorenzoni L, Ong P. *Price Setting and Price Regulation in Health Care*. OECD 2019. doi:10.1787/ed3c16ff-en

10 Carroll N, Lord JC. The Growing Importance of Cost Accounting for Hospitals. *J Health Care Finance* 2016;**43**:172./pmc/articles/PMC6910125/ (accessed 7 Jul 2021).

11 García-Cornejo B, Pérez-Méndez JA. Influence of cost systems on efficiency. An analysis of Spanish hospitals using public national databases. *Revista de Contabilidad-Spanish Accounting Review* 2020;**23**:249–62. doi:10.6018/rcsar.365031

For peer review only - http://bmjopen.bmj.com/site/about/guidelines.xhtml

12 Barber SL, Lorenzoni L, Ong P. *Price Setting and Price Regulation in Health Care*. OECD 2019. doi:10.1787/ed3c16ff-en

13 Munn Z, Peters MDJ, Stern C, *et al.* Systematic review or scoping review? Guidance for authors when choosing between a systematic or scoping review approach. *BMC Medical Research Methodology* 2018;**18**:1–7. doi:10.1186/S12874-018-0611-X/TABLES/1

14 Stenberg K, Lauer JA, Gkountouras G, *et al.* Econometric estimation of WHO-CHOICE country-specific costs for inpatient and outpatient health service delivery. *Cost Effectiveness and Resource Allocation* 2018;**16**:11. doi:10.1186/s12962-018-0095-x

15 Barber SL, O'Dougherty S, Vinyals Torres L, *et al.* Other considerations than: how much will universal health coverage cost? *Bull World Health Organ* 2020;**98**. doi:10.2471/BLT.19.238915

16 Joint Learning Network. Costing of Health Services for Provider Payment: A Practical Manual. 2014.

17 Mathauer I, Wittenbecher F. Hospital payment systems based on diagnosis-related groups: experiences in low- and middle-income countries. *Bull World Health Organ* 2013;**91**:746-756A. doi:10.2471/BLT.12.115931

18 Bredenkamp C, Bales S, Kahur K. Transition to Diagnosis-Related Group (DRG) Payments for Health Lessons from Case Studies. 2020.

19 Dianingati RS, Riewpaiboon A. Unit cost analysis of medical service in asia: A systematic review. doi:10.29090/psa.2021.02.19.034

20 Prinja S, Chauhan AS, Rajsekhar K, *et al.* Addressing the Cost Data Gap for Universal Healthcare Coverage in India: A Call to Action. *Value in Health Regional Issues* 2020;**21**:226–9. doi:10.1016/J.VHRI.2019.11.003

21 National Health Authority. Journey from HBP 1.0 to HBP 2.0. 2019.

22 KPMG. Ayushman Bharat — A big leap towards Universal Health Coverage in India. 2019.

23 Chatterjee S, Levin C, Laxminarayan R. Unit Cost of Medical Services at Different Hospitals in India. *PLoS ONE* 2013;**8**:e69728. doi:10.1371/journal.pone.0069728

24 Jassim AL. Cost Measuring of Medical Services in a Hospital using Relative Value Units (RVUs) – based Process Costing System. *undefined* 2011.

25 Prinja S, Singh MP, Rajsekar K, *et al.* Translating Research to Policy: Setting Provider Payment Rates for Strategic Purchasing under India's National Publicly Financed Health Insurance Scheme. *Applied Health Economics and Health Policy* 2021;:1–18. doi:10.1007/s40258-020-00631-3

26 Ocharot L, 'Sriratanaban J', Ngamkiatphaisan. Sureerat' ', *et al.* Estimating uncompensated medical care cost as a result of adverse events in a university hospital in Thailand. *Asian Biomedicine* 2016;**10**:261–7.

27 Riewpaiboon A. Standard cost list for economic evaluation in Thailand. *Value in Health* 2012;**15**:A645–A645.http://www.embase.com/search/results?subaction=viewrecord&from=export&id=L70915622%5Cnhttp://dx.doi.org/10.1016/j.jval.2012.08.255

For peer review only

28 Minh H van, Phuong NK, Özaltın A, *et al.* Costing of commune health station visits for provider payment reform in Vietnam. *Global Public Health* 2015;**10**. doi:10.1080/17441692.2014.944929

29 Vo TQ, Chaikledkaew U, Minh H, *et al.* Development of Relative Value Units for Unit Cost analysis of Medical Services in Vietnam. *Asian Journal of Pharmaceutics* 2018;**12**:S19–26.

30 Vo TQ, Chaikledkaew U, Minh H, *et al.* Hospital cost analysis in developing countries: A methodological comparison in Vietnam. *Asian Journal of Pharmaceutics* 2018;**12**:S8–18.

31 Ministry of Health Republic of Indonesia. THE COSTS OF DELIVERING HEALTH SERVICES IN INDONESIA: REPORT ON A PROSPECTIVE SURVEY 2010-2011. 2012.

32 Dianingati RS, Riewpaiboon A, Youngkong S. Indonesia Hospital Cost Analysis: a Micro-Costing Approach. *Jurnal Kesehatan Masyarakat* 2019;**14**. doi:10.15294/kemas.v14i3.15627

33 Doshmangir L, Rashidian A, Kouhi F, *et al.* Setting health care services tariffs in Iran: half a century quest for a window of opportunity. *International Journal for Equity in Health* 2020;**19**. doi:10.1186/s12939-020-01224-1

34 Ghaffari S, Doran C, Wilson A, *et al.* Investigating DRG cost weights for hospitals in middle income countries. *The International Journal of Health Planning and Management* 2009;**24**:251–64. doi:10.1002/hpm.948

35 Rasiah D. Why Activity Based Costing (ABC) is still tagging behind the traditional costing in Malaysia? *Journal of Applied Finance & Banking* 2011;:83–106.

36 Ali Jadoo SA, Aljunid SM, Nur AM, *et al.* Development of MY-DRG casemix pharmacy service weights in UKM Medical Centre in Malaysia. *DARU Journal of Pharmaceutical Sciences* 2015;**23**. doi:10.1186/s40199-014-0075-4

37 Martin A. Cambodia Hospital Costing and Financial Management Study. 2012.

38 Jacobs B, Hui K, Lo V, *et al.* Costing for universal health coverage: insight into essential economic data from three provinces in Cambodia. *Health Economics Review* 2019;**9**:29. doi:10.1186/s13561-019-0246-6

39 Mathauer I. Setting health insurance remuneration rates of private providers in Kenya: the role of costing, challenges and implications. *The International Journal of Health Planning and Management* 2011;**26**. doi:10.1002/hpm.1038

40 Zhao C, Wang C, Shen C, *et al.* Diagnosis-related group (DRG)-based case-mix funding system, a promising alternative for fee for service payment in China. *BioScience Trends* 2018;**12**. doi:10.5582/bst.2017.01289

41 Prinja S, Bahuguna P, Singh MP, *et al.* Determining Price Weights for Differential Case-Based Payments under India's National Publicly Financed Health Insurance Program. Under review: 2022.

42 National Health Authority. Journey from HBP1.0 to HBP2.0. New Delhi: 2019.

For peer review only

Figures

Figure 1: PRISMA diagram

Figure 2: Number of papers by country breakdown and types of study

Figure 3. Number of papers explaining the tariff setting scheme by country

Figure 4. Number of papers by year of publication.

Figure 5 Summary of evidence on the tariff setting process for case-based hospital payment in national health insurance schemes

For peer review only

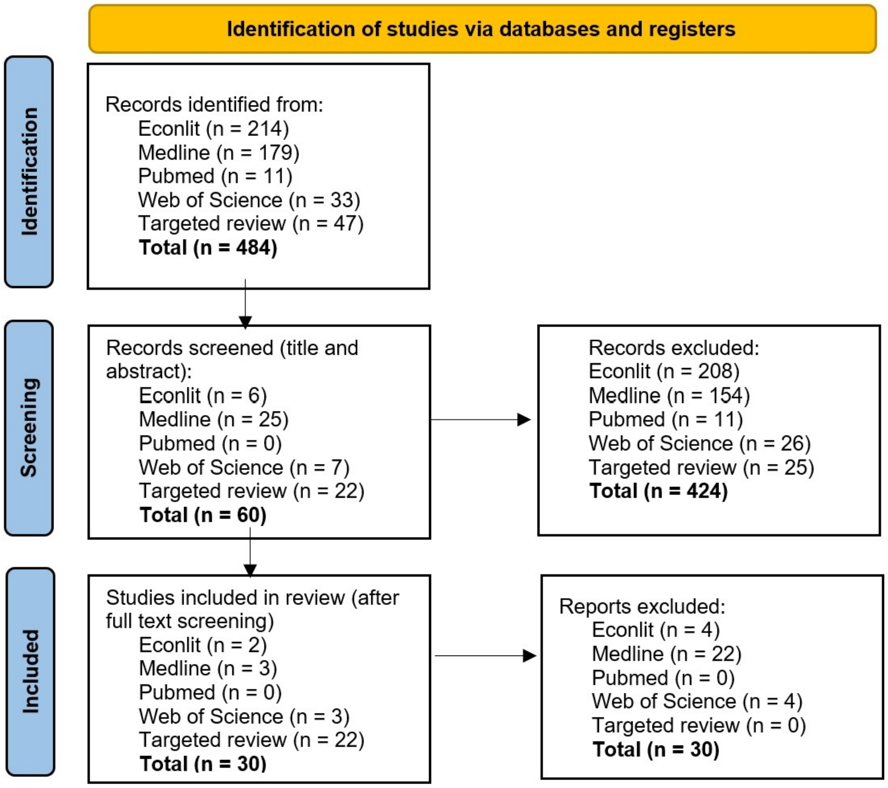

Figure 1 PRISMA diagram

109x92mm (288 x 288 DPI)

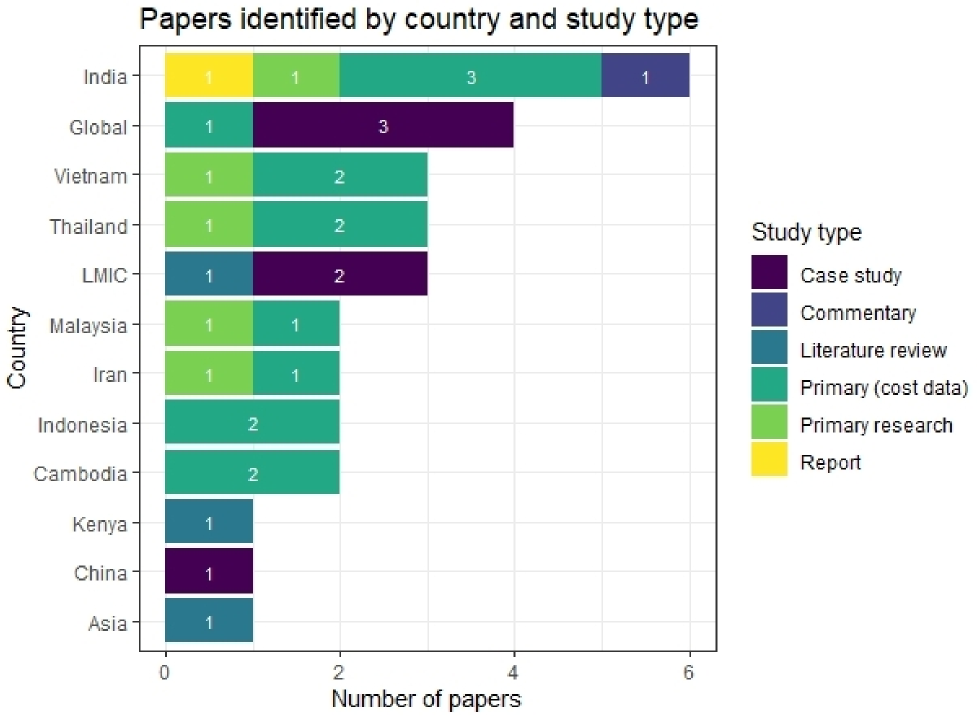

Figure 2: Number of papers by country breakdown and types of study

162x118mm (96 x 96 DPI)

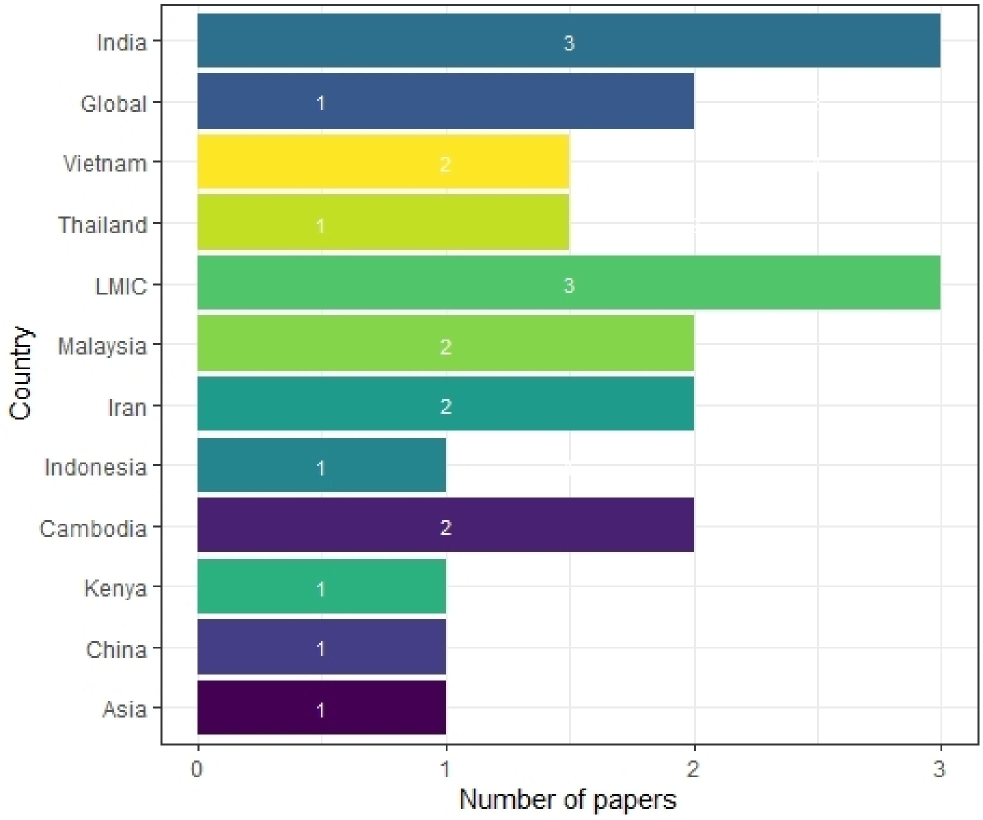

Figure 3. Number of papers explaining the tariff setting scheme by country

141x118mm (96 x 96 DPI)

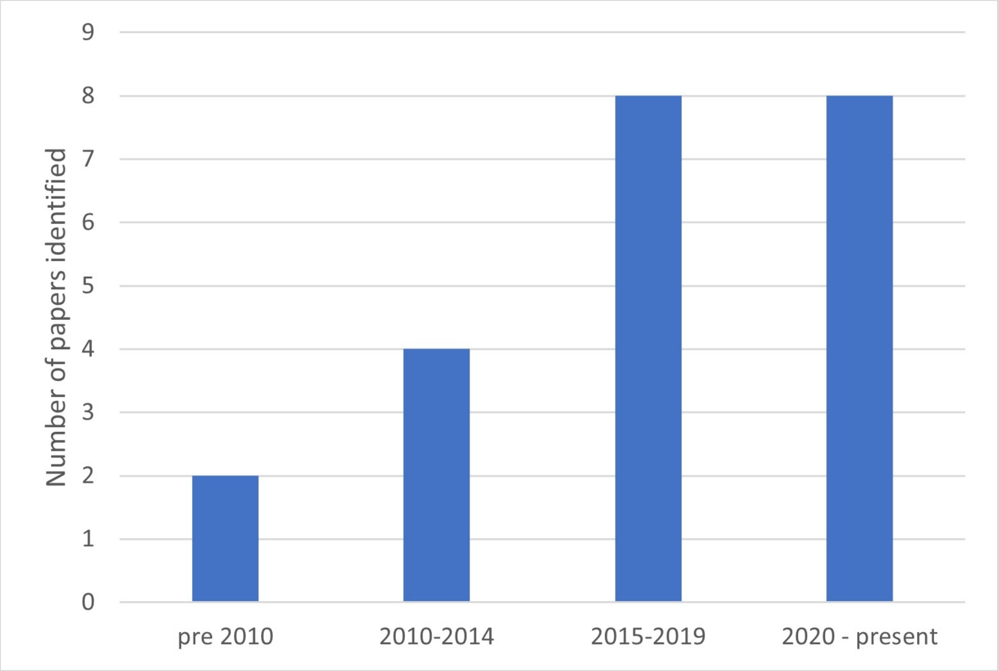

Figure 4. Number of papers by year of publication.

119x80mm (330 x 330 DPI)

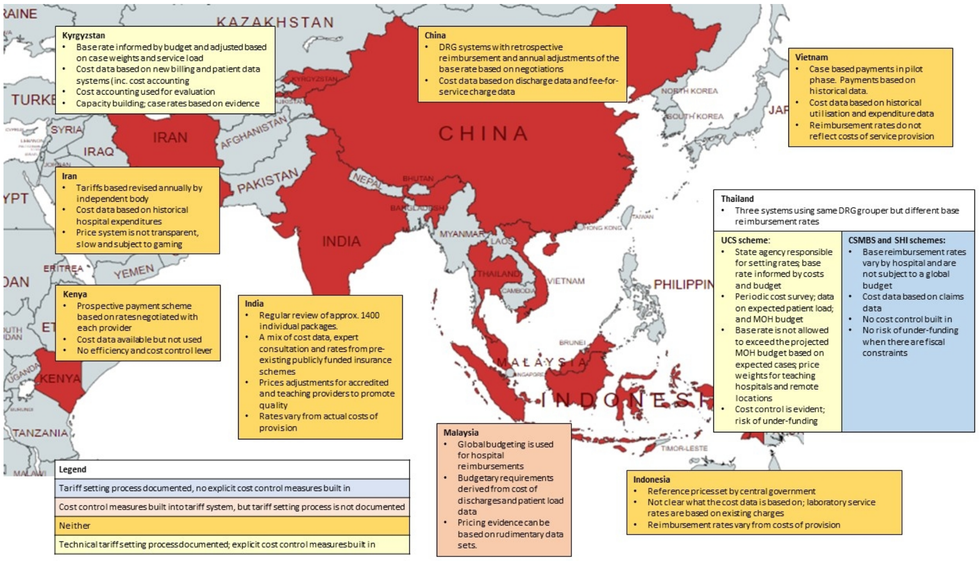

Figure 5 Summary of evidence on the tariff setting process for case-based hospital payment in national health insurance schemes

338x190mm (96 x 96 DPI)

Supplementary materials

Contents

Supplementary materials 1
Structured abstract 2
Table S1. Search strategy 4
Table S2. List of studies identified and included in the review 5
Table S3. Costing evidence for tariff setting in hospital payment schemes in LMICs 7

For peer review only

Structured abstract

Background.

Progress towards universal health coverage requires evidence based policy and price setting informed by good quality cost data systems. Establishing these systems can be complex and both resource and time intensive. and there is little documentary evidence of experiences building such systems in low-and-middle income countries (LMICs).

Objectives.

To synthesise evidence on the experiences of LMICs in institutionalizing cost data systems in order to derive lessons for the technical process of price-setting in the context of UHC goals.

Eligibility criteria.

Studies were included if they identified and/or described either the development of the national tariffs and/or the methods used to estimate or inform the tariffs for hospital services reimbursement.

Sources of evidence,

English-language publications since 2000 indexed in Pubmed, Medline, Econlit and the Web of Science.

Charting methods.

Papers were classified according to whether they explained the technical process of price-setting for reimbursements and whether they reported on the process of primary cost data collection for price-setting. We extracted information on cost data collection methods, outputs and commentary on how cost data was used as well as descriptions of the technical aspects of the tariff setting system and key strengths and challenges. A narrative review approach was used to summarise the evidence by country. Data extraction was performed by one reviewer and then checked by a second reviewer.

Results.

A total of 484 papers were initially identified of which 30 papers were considered eligible. Fourteen papers reported on primary cost data collection for price-setting purposes; 18 papers provided an

For peer review only

explanation of how cost evidence informs tariff-setting. Documented experience is largely focussed in the Asia region (n = 22) with countries at different stages of developing cost systems to inform tariff setting. Country experiences on healthcare cost accounting tend to showcase country costing experiences, methods and implementation. There is little documentation of how cost data has been incorporated into decision making and price setting. Where cost data, cost systems and costing has been used, improved transparency in decision making alongside increased service provision efficiency has followed.

Conclusions.

Countries need to build sustainable cost systems appropriate to their settings and budgets and adopt transparent processes and methodologies for translating costs into prices.

For peer review only

Table S1. Search strategy

Database	Search terms
**Web of Science**	(ALL=(("case*mix" or "cost systems" or "cost*accounting" or "ref*costs" or "resource weights" or "cost*weights" or "national reimbursement" or "DRG" or "hospital payment systems" or "fee*for*service"))) AND ALL=("LMIC" OR "low resource settings" OR "developing countries")
**Econlit**	("case*mix" or "cost systems" or "cost*accounting" or "ref*costs" or "resource weights" or "cost*weights" or "national reimbursement" or "DRG" or "hospital payment systems" or "fee*for*service").mp. [mp=heading words, abstract, title, country as subject]<br><br>No hits when combined with lmics
**Medline**	1. ("case mix" or "DRG" or "case based payment" or "hospital payment system" or "reimbursement" or "resource value unit" or "cost systems" or "cost accounting" or "reference costs" or "resource weights" or "cost weights" or "Price setting" or "service weights")<br>2. "Costs and Cost Analysis"/ or National Health Programs/ or diagnosis-related groups/ or hospitalization/<br>3. 1 OR 2<br>4. Developing Countries/<br>5. "universal health*<br>6. Universal Health Insurance/ or "Delivery of Health Care"/ or Insurance, Health/<br>7. 5 OR 6<br>8. 3 AND 4 AND 7
**PubMed**	("reference cost*" OR "reference price*") AND health* AND national* AND list*)<br>Since 2000

For peer review only

Table S2. List of studies identified and included in the review

Author	Year	Country	Aim of paper in relation to costing (rationale for inclusion)	Type of study	Cost data reported	Comments on country's cost system
Langebrunner JC et al	2009	LMIC (Kazakhstan, Kyrgyzstan)	Manual on methods for setting up provider based systems; provides methods and case study of costing accounting for case based payments	Case studies	Yes	Yes
Joint Learning Network	2014	LMIC	Costing specific resource for lmics	Case studies	Yes	Yes
Martin A	2012	Cambodia	Costing in Cambodian hospital	Primary (cost data)	Yes	Yes
Ministry of Health, Republic of Indonesia	2012	Indonesia	Report on a costing study for Indonesia	Primary (cost data)	Yes	Yes
Ghaffari S et al	2009	Iran	Costing for DRGs in Iran	Primary (cost data)	Yes	Yes
Mathauer I	2011	Kenya	Role of costing in setting insurance reimbursement rates in Kenya	Literature review	Yes	Yes
Jadoo SAA et al	2015	Malaysia	Documenting the development of DRG cost weights in pharmacy in Malaysia	Primary (cost data)	Yes	Yes
Dianingati JK et al	2019	Indonesia	Single site cost data collection to inform price setting	Primary (cost data)	Yes	Yes
Jacobs B et al	2019	Cambodia	Multiple site cost data collection to inform national policy including reimbursement rates	Primary (cost data)	Yes	Yes
Ocharot L et al	2016	Thailand	Cost implications of adverse events in DRG system	Primary (cost data)	Yes	Yes
Prinja S et al	2021	India	Comparing cost data with reimbursement rates in ABPMJAY, India	Primary (cost data)	Yes	Yes
Riewpaiboon, A. et al	2012	Thailand	Development of Relative Value Units for Unit Cost analysis of Medical Services in Thailand	Primary (cost data)	Yes	Yes
Vo TQ et al	2018a	Vietnam	Development of Relative Value Units for Unit Cost analysis of Medical Services in Vietnam	Primary (cost data)	Yes	Yes
Vo TQ et al	2018b	Vietnam	Comparison of hospital costing methods in Vietnam	Primary (cost data)	Yes	Yes
Dianingati RS et al	2021	Asia	Literature review of medical service costs in Asia	Literature review	Yes	No
Jassim AL et al	2011	India	Testing for RVU method for costing in hospitals in India	Primary (cost data)	Yes	No
Chatterjee S et al	2013	India	Hospital costing study	Primary (cost data)	Yes	No
Stenberg K et al	2018	Global	Estimating unit costs of health services at a country level based on global dataset	Primary (cost data)	Yes	-
Lian LL et al	2014	Taiwan	Assessing incentives in DRGs		No	Yes
Barber S et al	2019	Global (India, Malaysia, Thailand)	Manual and case studies on price setting for case-based payment	Case studies	No	Yes
Bredenkamp C et al	2020	Global (China, Kyrgyz Republic, Thailand)	Case studies of DRG transitions	Case studies	No	Yes

For peer review only

Author	Year	Country	Aim of paper in relation to costing (rationale for inclusion)	Type of study	Cost data reported	Comments on country's cost system
Zhao C et al	2018	China	Document China's experiences with shifting to case based payment schemes	Case studies	No	Yes
Prinja S et al	2020	India	Commentary on cost data for policy	Commentary	No	Yes
Mathauer I et al	2013	LMIC	Literature review of DRG experiences in LMICs	Literature review	No	Yes
Doshmangir L et al	2020	Iran	To document Iran's experience of tariff setting	Primary	No	Yes
Hoang VM, et al	2014	Vietnam	Reporting on costing in Vietnam for provider payment reform	Primary	No	Yes
National Health Authority	2019	India	Describes process of updating HBP package rates	Primary	No	Yes
Patcharanarumol K et al	2018	Thailand	Comparing strategic purchasing in two financing schemes	Primary	No	Yes
Rasiah, D et al	2011	Malaysia	Comparing methods for costing of health services in Malaysia	Primary	No	Yes
KPMG	2019	India	Overview of AB-PMJAY reform and financing mechanisms	Report	No	Yes
Barber S et al	2020	Global	To provide policy recommendations on estimating the cost of UHC	Case studies	No	Yes
Wagstaff A et al	2007	East Asia	Lessons learned in Asia for financing reforms including provider payment	Primary	No	Yes
Hu S et al	2008	China	Commentary	Commentary	No	No
Zeng W et al	2018	Global	Examining role of PBF in strengthening health systems	Commentary	No	No
Beck E et al	2012	LMIC	Describes the financial information required by policy makers and other stakeholders to enable them to make evidence-informed decisions and reviews the quantity and quality of the financial information available,	Literature review	No	No
Zou K et al	2020	China	Literature review of impact of case based payments in China	Literature review	No	No
Bertram M et al	2017	Global	Estimating unit costs for disease control programmes	Primary	No	No
Immunisation Costing Action Network	2018	LMIC	Costing of immunisation programmes	Primary	No	No
Jian W et al	2016	China	Assessing capacity of information system to implement DRGs (not cost system)	Primary	No	No
Watkins D et al	2020	LMIC	Resource requirement estimation of model health benefit packages (essential services)	Primary	No	No

For peer review only

Table S3. Costing evidence for tariff setting in hospital payment schemes in LMICs

Year	Author	Country/ Region	Type of study	Method of cost data collection	Generated output (what form does the cost data take e.g. Unit costs, DRG cost weights, base rates)	What is/was the cost data used for or what is the intended use
2021	Prinja S et al	India	Primary (cost data)	Step down allocation; mix of top down and bottom-up costing	Reference costs to inform price setting and HTA	Comparison of health benefit package reimbursement rates with costs; use in HTA
2021	Dianingati RS et al	Asia	Literature review	Methods used in hospital cost analysis in Asia include direct allocation, step-down allocation, simultaneous equation allocations, micro costing and simplified activity-based costing.	Different final outputs include average costs, patient level (micro) costs, RVUs and RCCs.	Not reported for the studies in the review
2019	Jacobs B et al	Cambodia	Primary (cost data)	Step down allocation; bottom-up costing	Costs of health services	Initial phase of establishing a routine costing system for health services
2019	Dianingati JK et al	Indonesia	Primary (cost data)	Step down allocation: micro costing	Unit cost of the laboratory services of a district hospital in Indonesia	To compare actual costs of laboratory services with government established reference prices
2018a	Vo TQ et al	Vietnam	Primary (cost data)	Step down allocation; Micro-costing	Relative value units (RVUs) for hospital services	Develop a set of RVUs for hospital services in Vietnam
2018b	Vo TQ et al	Vietnam	Primary (cost data)	1. Step down allocation; Micro-costing. 2. Hospital charges	Relative value units (RVUs) for hospital services	Comparison of different methods and identification of best method for the estimation of RVUs
2018	Stenberg K et al	Global	Primary	WHO-CHOICE service delivery unit costs based on regression analysis of country data sets	Cost per bed-day and cost per outpatient visit	Address cost information gap by producing standardised cost estimates that are comparable across countries
2016	Ocharot L et al	Thailand	Primary (cost data)	Hospital charges	Uncompensated adverse event costs for hospitalised patients	To demonstrate the cost implications of patient safety
2015	Jadoo SAA et al	Malaysia	Primary (cost data)	Step down allocation; bottom-up costing	DRG cost weights for pharmacy services	To identify the actual cost of pharmacy services by DRG case-mix group and inform reimbursement rates
2014	JLN	India Aarogyasri Hospital	Case study	Top down, direct and indirect costing for operating and capital costs.<br>For the cost of benefits packages a bottom-up approach was used. (4 hospitals; 42 procedures)	Costs of benefit packages	To set rates for 938 new benefit packages and revise estimates for pre-existing packages

For peer review only

Year	Author	Country/ Region	Type of study	Method of cost data collection	Generated output (what form does the cost data take e.g. Unit costs, DRG cost weights, base rates)	What is/was the cost data used for or what is the intended use
2014	JLN	Indonesia casemix	Case study	Top-down costing (137 hospitals)	Not specified	To estimate the cost of health services and construct cost weights for case based payments.
2014	JLN	Indonesia Health Facility	Case study	Top-down approach for both recurrent costs and capital costs. Bottom up approach to cost specific episodes of illness (200 hospitals)	Unit costs and episodes of illness	To estimate the production cost of services and drivers of cost variation among providers Capitation payments
2014	JLN	Central Asian Republic	Case study	Predominantly top down, but also bottom up to obtain allocation statistics (15 hospitals)	Cost per bed-day by department	To estimate the cost per bed-day by cost centres to inform DRG weight coefficients<br><br>There was sufficient data available for top down costing which could be conducted in a relatively short period of time
2014	JLN	Malaysian DRG	Case study	Top-down approach to measure and value personnel, drugs/medical supplies, overheads, and capital resource use. Bottom up planned for ICU stays, laboratory tests and radiological interventions. (10 hospitals)		To determine the cost of delivering health services in government hospitals to inform budgetary requirements<br><br>Top down approach was used most of the time, but for items that were heterogenous in their resource use or expensive a bottom up approach was more suitable
2014	JLN	India (PHFI)	Case study	Mixed method approach. Top down for the cost of resources consumed. Bottom up for personnel. (5 hospitals)	JLN costing exercise	PHFI used a mixed-method approach because data on resource use were not always available at the department level
2014	JLN	Phillipines	Case study	Analysis of claims. All tertiary hospitals.		Cost of health services and specific disease categories
2013	Chatterjee S et al	India	Primary (cost data)	Step down allocation; bottom up costing	Cost per OP visit, cost per bed day; cost per emergency visit, cost per OT case	Informs hospital administrators, to improve efficiency and demonstrate the feasibility of hospital cost analysis in India

For peer review only

Year	Author	Country/ Region	Type of study	Method of cost data collection	Generated output (what form does the cost data take e.g. Unit costs, DRG cost weights, base rates)	What is/was the cost data used for or what is the intended use
2012	Riewpaiboon A.	Thailand	Primary (cost data)	1. Reimbursement price list; 2. Bottom up/step down allocation; 3. Patient interviews	1. RVUs; 2. inpatient base rate; 3. patient non-medical costs	To develop a list of standard unit costs of medical services for Thailand for HTA.
2012	Ministry of Health, Republic of Indonesia	Indonesia	Primary (cost data)	Step-down accounting methodology	For hospital services: cost per outpatient, cost per inpatient and cost per bed-day	To better understand the cost of delivering health services across the country and inform policy on geographic resource allocation, the development of hospital payment system and capitation formulae.
2012	Martin A	Cambodia	Primary (cost data)	Top down costing/step-down cost accounting (10 hospitals)	Average cost per discharge, per inpatient data and per outpatient visit by hospital department	To link costs with funding sources and document use of funds as well as inform the revision of the National Charter on Health Financing including provider payment reforms.
2011	Mathauer I	Kenya	Literature review	Most recent, multi-site costing studies<br>1. Bottom up, ingredients costing from (11 faith-based provider hospitals -); 2. Step down cost accounting model (22 private for profit hospitals)	Cost per case (surgical, non-surgical and outpatients); cost per bed-day (surgical and non-surgical)	Costing information to inform resetting health insurance remuneration rates
2011	Jassim AL et al	India	Primary (cost data)	Step down allocation; process costing	Cost per relative value unit in support cost centres (e.g. laboratory)	To improve costing accuracy and provide guidelines for improved costing at the hospital level
2009	Ghaffari S et al	Iran	Primary (cost data); cost modelling	Primary: Step down allocation; ABC costing<br>Cost modelling: DRG cost weights imported from Australia	Relative value units, DRG costs and cost weights using indirect costs, direct care services and costs of care	To guide costing efforts for case mix funding models and identify optimal method for costing given data constraints.
2009	Langebrunner JC et al	Kyrgyzstan	Case studies	Cost accounting: Step down cost allocation to departmental level, Adapted from US medicare cost reports (initially 1 public hospital)	Cost per bed day and cost per case	Informed case base payment rates in DRG system

For peer review only

Preferred Reporting Items for Systematic reviews and Meta-Analyses extension for Scoping Reviews (PRISMA-ScR) Checklist

SECTION	ITEM	PRISMA-ScR CHECKLIST ITEM	REPORTED ON PAGE #
**TITLE**			
Title	1	Identify the report as a scoping review.	Yes
**ABSTRACT**			
Structured summary	2	Provide a structured summary that includes (as applicable): background, objectives, eligibility criteria, sources of evidence, charting methods, results, and conclusions that relate to the review questions and objectives.	Supplement
**INTRODUCTION**			
Rationale	3	Describe the rationale for the review in the context of what is already known. Explain why the review questions/objectives lend themselves to a scoping review approach.	1-2
Objectives	4	Provide an explicit statement of the questions and objectives being addressed with reference to their key elements (e.g., population or participants, concepts, and context) or other relevant key elements used to conceptualize the review questions and/or objectives.	1,4-6
**METHODS**			
Protocol and registration	5	Indicate whether a review protocol exists; state if and where it can be accessed (e.g., a Web address); and if available, provide registration information, including the registration number.	Doesn't exist
Eligibility criteria	6	Specify characteristics of the sources of evidence used as eligibility criteria (e.g., years considered, language, and publication status), and provide a rationale.	6-7
Information sources*	7	Describe all information sources in the search (e.g., databases with dates of coverage and contact with authors to identify additional sources), as well as the date the most recent search was executed.	6
Search	8	Present the full electronic search strategy for at least 1 database, including any limits used, such that it could be repeated.	Supplement
Selection of sources of evidence†	9	State the process for selecting sources of evidence (i.e., screening and eligibility) included in the scoping review.	6-7
Data charting process‡	10	Describe the methods of charting data from the included sources of evidence (e.g., calibrated forms or forms that have been tested by the team before their use, and whether data charting was done independently or in duplicate) and any processes for obtaining and confirming data from investigators.	7
Data items	11	List and define all variables for which data were sought and any assumptions and simplifications made.	Qualitative themes identified – pg 7

For peer review only - http://bmjopen.bmj.com/site/about/guidelines.xhtml

SECTION	ITEM	PRISMA-ScR CHECKLIST ITEM	REPORTED ON PAGE #
Critical appraisal of individual sources of evidence§	12	If done, provide a rationale for conducting a critical appraisal of included sources of evidence; describe the methods used and how this information was used in any data synthesis (if appropriate).	Not done
Synthesis of results	13	Describe the methods of handling and summarizing the data that were charted.	7
**RESULTS**			
Selection of sources of evidence	14	Give numbers of sources of evidence screened, assessed for eligibility, and included in the review, with reasons for exclusions at each stage, ideally using a flow diagram.	Figure 1 and pg 7-8
Characteristics of sources of evidence	15	For each source of evidence, present characteristics for which data were charted and provide the citations.	Table 1 and supplement and pg 7-8
Critical appraisal within sources of evidence	16	If done, present data on critical appraisal of included sources of evidence (see item 12).	Not done
Results of individual sources of evidence	17	For each included source of evidence, present the relevant data that were charted that relate to the review questions and objectives.	Table 1 and supplement
Synthesis of results	18	Summarize and/or present the charting results as they relate to the review questions and objectives.	Table 1 and pgs 8-11
**DISCUSSION**			
Summary of evidence	19	Summarize the main results (including an overview of concepts, themes, and types of evidence available), link to the review questions and objectives, and consider the relevance to key groups.	11-13
Limitations	20	Discuss the limitations of the scoping review process.	13-14
Conclusions	21	Provide a general interpretation of the results with respect to the review questions and objectives, as well as potential implications and/or next steps.	14
**FUNDING**			
Funding	22	Describe sources of funding for the included sources of evidence, as well as sources of funding for the scoping review. Describe the role of the funders of the scoping review.	15

JBI = Joanna Briggs Institute; PRISMA-ScR = Preferred Reporting Items for Systematic reviews and Meta-Analyses extension for Scoping Reviews.
* Where *sources of evidence* (see second footnote) are compiled from, such as bibliographic databases, social media platforms, and Web sites.
† A more inclusive/heterogeneous term used to account for the different types of evidence or data sources (e.g., quantitative and/or qualitative research, expert opinion, and policy documents) that may be eligible in a scoping review as opposed to only studies. This is not to be confused with *information sources* (see first footnote).
‡ The frameworks by Arksey and O'Malley (6) and Levac and colleagues (7) and the JBI guidance (4, 5) refer to the process of data extraction in a scoping review as data charting.
§ The process of systematically examining research evidence to assess its validity, results, and relevance before using it to inform a decision. This term is used for items 12 and 19 instead of "risk of bias" (which is more applicable to systematic reviews of interventions) to include and acknowledge the various sources of evidence that may be used in a scoping review (e.g., quantitative and/or qualitative research, expert opinion, and policy document).

From: Tricco AC, Lillie E, Zarin W, O'Brien KK, Colquhoun H, Levac D, et al. PRISMA Extension for Scoping Reviews (PRISMAScR): Checklist and Explanation. Ann Intern Med. 2018;169:467–473. doi: 10.7326/M18-0850.

For peer review only - http://bmjopen.bmj.com/site/about/guidelines.xhtml

BMJ Open

The role of healthcare cost accounting in pricing and reimbursement in low- and middle-income countries: a scoping review

Journal:	*BMJ Open*
Manuscript ID	bmjopen-2022-065019.R1
Article Type:	Original research
Date Submitted by the Author:	13-Sep-2022
Complete List of Authors:	GUINNESS, LORNA; Center for Global Development<br>Ghosh, Srobana; Center for Global Development<br>Mehndiratta, Abha; Center for Global Development<br>Shah, Hiral A.; Imperial College London, Department of Infectious Disease Epidemiology; Center for Global Development
**Primary Subject Heading**:	Health economics
Secondary Subject Heading:	Health policy, Health services research, Health economics, Health informatics
Keywords:	Health economics < HEALTH SERVICES ADMINISTRATION & MANAGEMENT, Health policy < HEALTH SERVICES ADMINISTRATION & MANAGEMENT, International health services < HEALTH SERVICES ADMINISTRATION & MANAGEMENT

SCHOLARONE™
Manuscripts

I, the Submitting Author has the right to grant and does grant on behalf of all authors of the Work (as defined in the below author licence), an exclusive licence and/or a non-exclusive licence for contributions from authors who are: i) UK Crown employees; ii) where BMJ has agreed a CC-BY licence shall apply, and/or iii) in accordance with the terms applicable for US Federal Government officers or employees acting as part of their official duties; on a worldwide, perpetual, irrevocable, royalty-free basis to BMJ Publishing Group Ltd ("BMJ") its licensees and where the relevant Journal is co-owned by BMJ to the co-owners of the Journal, to publish the Work in this journal and any other BMJ products and to exploit all rights, as set out in our licence.

The Submitting Author accepts and understands that any supply made under these terms is made by BMJ to the Submitting Author unless you are acting as an employee on behalf of your employer or a postgraduate student of an affiliated institution which is paying any applicable article publishing charge ("APC") for Open Access articles. Where the Submitting Author wishes to make the Work available on an Open Access basis (and intends to pay the relevant APC), the terms of reuse of such Open Access shall be governed by a Creative Commons licence – details of these licences and which Creative Commons licence will apply to this Work are set out in our licence referred to above.

Other than as permitted in any relevant BMJ Author's Self Archiving Policies, I confirm this Work has not been accepted for publication elsewhere, is not being considered for publication elsewhere and does not duplicate material already published. I confirm all authors consent to publication of this Work and authorise the granting of this licence.

For peer review only

The role of healthcare cost accounting in pricing and reimbursement in low- and middle-income countries: a scoping review

Lorna Guinness (ORCID -0000-0002-1013-4200), PhD, Center for Global Development, London, United Kingdom

Srobana Ghosh, (ORCID 0000-0002-9588-0973) MSc, Center for Global Development, London, United Kingdom

Abha Mehndiratta, MBBS, DCH, DNB, MPH, Center for Global Development, London, United Kingdom

Hiral Anil Shah (ORCID - 0000-0003-0204-451X), MRC Center for Global Infectious Disease Analysis, Department of Infectious Disease Epidemiology, School of Public Health, Imperial College London, London, UK.

Corresponding author

Lorna Guinness
Great Peter House,
Abbey Gardens,
Great College St,
London
SW1P 3SE
Email: lornaguinness@gmail.com
Phone: +447926674052

Word count:

4074

For peer review only - http://bmjopen.bmj.com/site/about/guidelines.xhtml

Abstract

Objectives: Progress towards universal health coverage (UHC) requires evidence based policy including good quality cost data systems. Establishing these systems can be complex, resource intensive and take time. This study synthesises evidence on the experiences of low-and-middle income countries (LMICs) in the institutionalisation of cost data systems to derive lessons for the technical process of price-setting in the context of UHC.

Design: A scoping review and narrative synthesis of publicly available information

Data sources: Pubmed, Medline, Econlit, the Web of Science and grey literature searched from January 2000 to April 2021.

Eligibility Criteria: English-language papers published since 2000 that identified and/or described development of and/or methods used to estimate or inform national tariffs for hospital reimbursement in LMICs. Papers were screened by 2 independent reviewers.

Data extraction and synthesis: Extraction was performed by one reviewer and checked by the second reviewer on: the method and outputs of cost data collection; commentary on the use of cost data; description of the technical process of tariff setting; and strengths and challenges of the approach. Evidence was summarised using narrative review.

Results: Thirty of 484 papers identified were eligible. Fourteen papers reported on primary cost data collection; 18 papers explained how cost evidence informs tariff-setting. Experience was focused in Asia (n = 22) with countries at different stages of developing cost systems. Experiences on cost accounting tend to showcase country costing experiences, methods and implementation. There is little documentation how data has been incorporated into decision making and price-setting. Where cost information and cost systems was used, improved transparency in decision making alongside increased efficiency has followed.

Conclusions

For peer review only

For peer review only - http://bmjopen.bmj.com/site/about/guidelines.xhtml

There are widely used and accepted methods for generating cost information. Countries need to build sustainable cost systems appropriate to their settings and budgets and adopt transparent processes and methodologies for translating costs into prices.

STRENGTHS AND LIMITATIONS OF THIS STUDY

- The review relied on publicly available information and there may be further information that was not accessible.
- The terminology on the role of cost evidence in price-setting in the literature is poorly defined and inconsistent.
- Our findings are limited by the inadequate documentation on how cost data and price evidence is used in the price-setting process in LMICs.
- The review explored both costing methods used in cost systems and how cost information is used to inform price-setting.

For peer review only

For peer review only - http://bmjopen.bmj.com/site/about/guidelines.xhtml

INTRODUCTION

Low-and-middle income countries (LMICs) have been making significant progress towards universal health coverage (UHC) through innovative healthcare financing. One focus of healthcare financing reforms has been reimbursement schemes that target the explicit goals of efficiency and cost containment while improving quality and reaching the poor and vulnerable. Historically, block grants have been used to reimburse healthcare providers in publicly financed systems in LMICs. However, as national-level public purchasers have evolved and a broader range of healthcare providers (e.g. private or faith-based healthcare providers) are accepted as part of the developing health system, newer prospective payment mechanisms and systems of provider reimbursement are being used by government purchasers of healthcare [1].

Common prospective payment mechanisms such as case-based payments for the reimbursement of secondary and/or tertiary care and capitation payments for primary care providers are now being championed across developing regions and countries. Case-based payments are equivalent to a system where providers are reimbursed based on cases treated rather than per service or per bed days [2]. On the other hand, capitation based payments are equivalent to a payment system where lump-sum payments are made to care providers based on the number of patients in a target population [2,3].

Setting reimbursement rates requires a reliable cost evidence base to enable price negotiations that are transparent, facilitate cost control and help drive providers to more efficient services. In principle, information is needed on the average cost per case across all admissions and/or visits (a base rate) and the relative value of different conditions as classified in the respective country (e.g., Diagnosis Related Groups (DRGs), specialty-based classification, intervention specific health benefit package etc) [4–6]. In a case-based payment scheme, the service groups are often DRGs or a similar grouping system that provides a means of relating the type of patients a hospital treats to the costs incurred by the hospital. For capitation-based systems the grouping is related to the average expected cost of treating a patient under the care of the provider. In both types of system, the technical process of price-setting requires a robust cost system to be in place, using principles that can be guided or even mandated by a purchaser, in order to generate reliable health service cost estimates.

For peer review only - http://bmjopen.bmj.com/site/about/guidelines.xhtml

Raulinajtys-Grzybek (2014) defines the cost system as "*a cost accounting system that ensure the cost homogeneity of individual groups* (of services)" [7]. There are however variation in costing systems across health systems as a result of choices about the process of collecting and verifying the data, the stage of development of the reimbursement system, the regulation around cost accounting and the costing methodology used [7]. For example, they can vary from one off costing studies to regular national costing surveillance [7,8]. Some cost surveys involve all participating providers e.g., in the UK and Australia all providers are mandated to submit cost accounting information; in others, only a sample of representative providers is used e.g. France, Germany and Thailand [9].

In terms of costing methodology, according to Gapenski and Reiter (2016) "the holy grail of cost estimation is costing at the service or individual patient level" [10]. More advanced systems e.g., those in the UK and Australia use bottom-up style costing methods to derive patient level DRG costs [11]; but there are simplified methods available that calculate the average cost of procedure through step down allocation methods [12]. Whichever approach is taken, it is important that the costing is nationally acceptable and can capture structural differences in cost that might be present (types of provider, demography, geography etc) as well as variability between the cost of the conditions treated. In addition, the national costing system should be standardised across providers, creating transparency and comparability [8].

In LMICs, while the process of payment reform has been well documented, there is less information available about the role of cost information in the technical process of setting reimbursement rates. An increasing number of countries are moving towards case-based payment schemes for secondary care within their UHC strategies. Documenting the cost systems used to generate evidence for rate setting can provide lessons for the further development of existing systems or the establishment of new ones. The aim of this paper is therefore to synthesise the evidence on the role of cost accounting in setting reimbursement rates for case-based payment schemes in LMICs. We performed a scoping review and narrative synthesis to document the current practice in LMICs based on publicly available information and recommend steps for the technical process of price-setting in LMICS in the context of UHC goals.

For peer review only - http://bmjopen.bmj.com/site/about/guidelines.xhtml

METHODS

Search Strategy and Selection Process

A scoping review approach was used to synthesise the evidence on cost accounting in LMICs. We aimed to map the body of literature, clarify key concepts and identify any gaps in the research [13]. We further refined our research question using a standard PICO framework:

- Problem: technical process for price-setting for hospital case-based payments in LMICs
- Intervention: cost systems
- Comparator: non-cost-based methods
- Outcome: improved cost evidence base for price-setting

We used several approaches in identifying the literature. First, we conducted a search of the literature for peer-reviewed English-language publications indexed in Pubmed, Medline, Econlit and in the Web of Science on the subject of national level health system costing in LMICs and the associated design of their costing systems. Our search was conducted using the following terms: ("case*mix" or "cost systems" or "cost*accounting" or "ref*costs" or "resource weights" or "cost*weights" or "national reimbursement" or "DRG" or "hospital payment systems" or "fee*for*service") AND ("LMIC" or "low resource settings" or "developing countries"). We conducted a search that included the country name of all LMICs, as defined by the World Bank. To complement this, we consulted existing libraries of both grey and peer-reviewed literature held by the research team. We then conducted an analysis of text words contained in the title and abstract to help identify further keywords and index terms. A further search was then conducted using the identified keywords and index terms. Finally, the reference list of all identified reports and articles were reviewed for any reports or papers that might have been missed. The search strategy is provided in the Supplement (Table S1).

Eligibility Criteria & Screening

Papers in the English language were included. We searched for literature published between January 2000 and April 2021. We restricted the search to this time period as, in LMICs, case-based payments

For peer review only - http://bmjopen.bmj.com/site/about/guidelines.xhtml

in national insurance programmes are a relatively new phenomenon and the quality and use of cost data was very limited prior to this [14,15]. Results were hand screened to ensure that the topic was limited to the eligible countries (LMICs as defined by the World Bank) and that the study identified and/or described the development of the national tariffs for hospital reimbursements and/or the methods used to estimate or inform the tariffs for hospital services reimbursement. The titles and abstracts were screened independently by two reviewers (SG, LG) as per the inclusion and exclusion criteria defined by the study. The second screen involved reviewing full texts.

Data Extraction & Synthesis

The papers were then classified according to whether they explained the technical process of price-setting for reimbursements (i.e., if and how cost data was used) and whether they reported on the process of primary cost data collection for price-setting. For those papers or case studies reporting on the process of primary cost data collection for price-setting, we extracted information on the method of cost data collection, the output and any commentary on how the cost data was used for price-setting for hospital case-based payments including identifying the commissioning agency. From the papers that described how cost data is used in price-setting, we extracted information on any description of the technical aspects of the tariff setting system in place, at the time of the study, and the key strengths and challenges of the approach used. For those papers describing more than one country experience, only evidence on LMIC experiences was extracted. We use a narrative review approach to summarise the evidence by country. Data extraction was performed by one reviewer (SG) and then checked independently by another reviewer (LG).

Patient and Public Involvement

Neither patients or public were involved in the design, conduct, reporting or dissemination plans of our research.

For peer review only

For peer review only - http://bmjopen.bmj.com/site/about/guidelines.xhtml

RESULTS

Overview of the literature

A total of 484 papers were initially identified of which 424 papers were excluded in the initial screening. The second screen involved reviewing full texts, leading to the inclusion of 30 papers in the review as described in the PRISMA diagram in Figure 1.

Of the 30 papers extracted (see Supplement Table S2), 7 papers stated a global focus (including LMICs) [1,4,16–20] and one paper reported to be focused on the Asia region [21] (see Figure 2). Of the single country focused papers, 6 related to India [22–27]. We found 3 studies each related to Thailand [6,28,29] and Vietnam [30–32]. There were 2 studies focused on each of Indonesia [33,34], Iran [35,36], Malaysia [37,38] and Cambodia [39,40] and one study each for Kenya [41] and China [42]. Further, within the global papers, we identified case studies on: Kazakhstan, Kyrgyzstan, India, Malaysia, Thailand, and China [1,4,16–20].

Papers reporting on primary collection of cost data to inform tariff-setting

Twenty-three case studies from 14 studies reported on primary cost data collection for price-setting purposes in a single country setting, either describing methods or both methods and results (see Supplement Table S3). Twelve case studies also had the explicit aim of generating cost information for broader policy processes. In terms of pricing, two case studies reported on a costing exercise that was designed to inform capitation payment rates [18,33], six studies aimed at generating cost weights for DRGs [4,18,38][1] or unspecified case groups [18,29,31][2] and 3 studies reported on the estimation of the costs of health benefit packages [18,27][3]. A final case study reviewed the available cost evidence for informing price-setting in the National Health Insurance Fund, Kenya [41].

For the studies reporting costs, cost per service unit at the hospital level was the most frequently sited output e.g., cost per bed day, cost per admission and cost per outpatient visit. Three studies generated unit costs for specific services: cost per adverse event [28]; laboratory services [34]; and pharmacy

[1] Joint Learning Network case studies: Central Asian Republics
[2] Joint Learning Network case studies: Indonesia Ministry of Health
[3] Joint Learning Network case studies: PhilHealth and India Aaorgyasri

For peer review only - http://bmjopen.bmj.com/site/about/guidelines.xhtml

services [38]. A further three case studies generated costs of health benefit packages [18,27][3]. Relative value units (RVU's) were the primary output of 7 studies [18,26,29,31,32,36,38], one of which also explicitly estimated an inpatient base rate [29].

Fourteen of the case studies were commissioned by the local ministry of health or agency acting on their behalf. However, in many cases, it was not clear who had commissioned the costing or if the study was linked to the national policy process [16,18,25,26,28,31,32,38][4]. Two studies evaluated different methods for generating robust relative value units [32,36].

Papers reporting on how cost data informs the tariff-setting process

We identified 18 papers that provided explanation of the technical process of tariff-setting, documenting experiences in 10 different countries (see Figure 3). Eight papers were published since the beginning of 2020, 8 papers in the period 2015-2019, 4 papers in the period 2010-2014, and 2 papers were published before 2010 (see Figure 4). The papers provided mixed levels of detail on the technical processes of price-setting and the strengths and weaknesses in each locality.

The current tariff system, presence of an explanation of the price-setting process, the data used in price-setting and resulting policy levers and implications are summarised by country in Figure 5. Only one study described tariff setting in Africa [41]. The paper reviewed the available evidence on costs for informing Kenya's National Health Insurance Fund prices and was published in 2011. Although the cost information were considered reliable by all stakeholders, in part due to their involvement in the costing exercises, the costs had not been used for setting prices at the time of the study.

The other countries covered were all in Asia. In the Central Asia region, three papers focus on the reform of the tariff setting system in Kyrgyzstan [4,17,20]. The technical process of price-setting is clearly documented. This process includes cost control measures derived from linking the reimbursement rates to the ministry of health budget. The Thai Universal Coverage Scheme (UCS) also provides an example in which cost control is built into the base rate through linkages to the

[4] Joint Learning Network case studies: India – Public Health Foundation of India

For peer review only

budget. However, as figure 5 states, in Thailand, there are 3 government funded and implemented schemes. Although all Thai schemes use the same Thai-DRG grouper, the Civil Service Medical Benefit Scheme (CSMBS) and Social Health Insurance (SHI) schemes do not use cost control mechanisms, as the rates are not linked to an overall budget and are different rates for different hospitals [1,6,20,29].

The Kyrgyz reforms were found to be vulnerable to gaming as the system does not make full use of the data available potentially leading to misclassification of diagnoses. This potential for gaming is also highlighted in Iran. In contrast to the systematic introduction and use of cost accounting in Kyrgyzstan, Iran's price-setting process involves a technical assessment by an independent body but with limited transparency (see Figure 5) [35,36]. Doshmangir et al note that without an objective and explicit mechanism in the updating of medical tariff and no structure to effectively manage conflicts of interest, the pricing system has in effect become "a tool for revenue manipulation" [35].

Challenges also arise when reimbursement rates are not based on cost evidence. In India's national insurance programme for the poor and vulnerable, the government used existing information to set reimbursement rates while establishing a review system to allow for modification and improvement over time (see Figure 5) [17]. However, the method in which information from costing studies, experts and rates under previous schemes is compiled is not transparently reported on. Studies show that the rates vary considerably from actual costs (42% of HBPs had a price less than 50% of the true cost in 2018) [27] . This could affect the recruitment of providers, the coverage and quality of care, and bring the rates themselves into doubt (Prinja S et al "Determining Price Weights for Differential Case-Based Payments under India's National Publicly Financed Health Insurance Program",Unpublished, 2022). Indonesia faces a similar problem in respect of laboratory services. Dianingati et al, report on a lack of transparency in the development of the reference prices set by the government and that the true costs of service delivery are 40-53% higher than the reference price. However, validation of the rates is difficult as data on the cost of healthcare services is still limited to a few services, in focal geographical areas, restricted to the public sector with few published and readily accessible cost data analyses/ data sets [22,27].

For peer review only - http://bmjopen.bmj.com/site/about/guidelines.xhtml

Both the Thai UCS and Kyrgyz price-setting systems use cost accounting to inform their base rates and case weights. Langebrunner et al note how cost accounting has been used as an evaluation tool and allowed for tariff adjustments based on evidence so that payments match services in Kyrgyzstan. Similarly, in the Thai UCS scheme, a key feature of the tariff setting is the cost information on which pricing for the UCS is based. This is collected on a periodic basis in a cost survey and has evolved from initial work using an RVU method and "top level" hospital cost data [29] to a 900 hospital survey. While no study described how the system has reformed, the papers note that the gradual, step wise implementation allowed for institutional and technical capacity building.

There was less detail reported on the tariff setting process in China, Indonesia, Malaysia and Vietnam. The one study documenting tariff setting processes in China raises concerns that the schemes used retrospective payment system and fails to build in efficiency and cost control [20]. In Vietnam there was also concern that the fees and the payment schemes bore little relationship to the costs of delivering services, although the RVU method used to calculate rates for the capitation scheme was relatively simple. Similarly, while the Malaysian system was designed for global budgeting, it also demonstrates that pricing evidence can be based on skeletal data sets such as those that focus on large expenditure items and patient data that are feasible to collect [1].

DISCUSSION

Key findings

Our scoping review has explored the literature on using the cost evidence base for setting prices in national health insurance schemes in LMICs. It has identified a significant gap in the literature in this area. However, despite this, we found consistent themes around the need to use cost information using a systematic methodology, reporting this transparently and working with providers to develop the system. Our review confirms that cost evidence can increase efficiency of service provision by increasing the policy evidence base. To generate this evidence, countries need to build cost systems appropriate to the setting and data availability but allowing for and investing in increasing complexity as data systems improve. While national costing surveillance should be an aspiration, prices may be set using cost evidence from one off costing studies, or even hospital charges. The method in which

For peer review only - http://bmjopen.bmj.com/site/about/guidelines.xhtml

these data are then used to set base rates and price weights, especially in the absence of national surveillance, should be part of a transparent process that involves relevant stakeholders and takes account of heterogeneity in costs driven by demand side (e.g. condition or patient specific) and supply side (e.g. hospital location) factors.

Strengths and weaknesses of the scoping review

The scoping review found a limited level of evidence and many of the studies are old and may be outdated. In addition, early reforms were reported for some countries, and it was not possible to determine how the tariff-setting processes have evolved. For example, Vietnam's pilot study was published in 2014 but there were no corresponding papers documenting next steps; nor did we identify more recent reports on Kenya and Cambodia where costing evidence from large multi-site studies were identified.

A second limitation was the focus of the review on a single component of the tariff setting process which may have limited the evidence generated. During the screening process many articles were identified on the process of developing DRG type reforms, but few focused on the price-setting process and how cost evidence is used in the price-setting process. This was compounded by the terminology related to the role of cost evidence in price-setting in the literature which is poorly defined. The terms cost and price are used in many different ways to mean different things which may have led to some omissions. We addressed these issues by extending the search and performed additional searches using the key words identified in the initial papers found that met the inclusion criteria. Finally, our review of the grey literature was limited to a google search and snowballing from references that were identified in the initial search. It is likely evidence in this area lies in government and donor reports that we missed and restricting reports to the English language may have compounded this.

These challenges serve to highlight the lack of attention on this aspect of tariff-setting in the literature and the need for further research.

For peer review only

Implications of the findings for researchers and policymakers keen to establish cost systems

The key message from the review is that cost systems help create a transparent evidenced based process for price-setting. A centralised cost accounting system, such as was developed in Kyrgyzstan, was considered a major strength of the broader health system reforms – allowing for policy reform to anticipate expenditure needs and enabling the government to effect change more effectively. Leaving base rates open to negotiation at the individual provider level with minimal evidence on costs and efficiency of service provision, leaves the system vulnerable to gaming. Studies from Iran and Thailand emphasise how important the cost system is in the setting of health benefit package/ DRG prices, to minimise gaming and prevent cost escalation [6,35].

In addition, creating a tariff setting system that does not use costs based on empirical evidence can embed inefficiencies and possibly make it more difficult to implement costing in the future [35], further underlining the need for cost systems to generate good quality data, based on accepted methodologies. As well as cost data collection, a systematic method for translating costs into prices or reimbursement rates helps avoid skewed incentives within the prices, evident in the unexplained differences between costs and reimbursement rates found in India and Indonesia [27,34]. Langenbrunner's reporting of the Kyrgyzstan case study provides the most comprehensive description for the calculation of the base rate and case-based weightings and how to use these to set reimbursement rates [4]. Patharanarumol et al also describe the principles applied for estimating the base rates and weights in the Thai UCS scheme [6]. For both settings, explicitly accounting for the budget in the price estimation using an "economic adjustment" is a key mechanism of cost control. This level of transparency is not apparent elsewhere in the literature identified. For example, while the different strands of information used for price-setting are documented in the reports on India, the method for combining this information is not available [27,43]. In Iran, the lack of such a

For peer review only - http://bmjopen.bmj.com/site/about/guidelines.xhtml

methodology was reported as a significant problem for the DRG system as a whole leading to price manipulation by different stakeholders [35].

Methods for cost data collection also need to be appropriate for the setting. Studies from Thailand and Vietnam compare different approaches to obtaining the base rate and cost weights for health technology assessment and pricing. They compare micro costing with relative value unit approaches and find both to be feasible with micro-costing being highly resource intensive. The costing methods tend to follow the same principles using top-down allocation methods supplemented with bottom-up costing if resources allow, for some specific inputs. In Malaysia, one study demonstrated the feasibility of using the electronic prescribing system to generate DRG weights, although it was recognised that these were not available in most facilities.

It is also important to be aware of the trade off in accuracy and resources needed to generate the required cost information. Where resources are highly constrained, any data can be better than no data, particularly if the data are reported transparently and how the data informs decisions is clearly communicated and accounted for. If cost accounting is not the norm and the budget is limited, costing for price-setting may need to start with simpler methods, using, for example, expenditure data, relative value units and smaller samples of facilities. Alternative approaches, for example in India, Cambodia and Kenya, have started with the implementation of baseline multi-site costing studies. Although these are one off exercises, they provide an evidence base and good practice on which to build. The costing itself can also be a way to bring stakeholders into the price-setting process and build capacity for future costings. The example of Kyrgyzstan shows how implementing a cost system is a slow, gradual and complex process. The established costing systems identified in the literature illustrate how a cost system has evolved from one-off exercises and developed into complex system with increasing numbers of participating providers (Thailand, Kyrgyzstan, China).

Future research

Our review is the first study in this area for LMIC settings, providing a foundation upon which further evidence in this area can be developed. More work is required to document better the practice of cost data collection, the costing methods used for informing national tariffs and how cost information is

For peer review only - http://bmjopen.bmj.com/site/about/guidelines.xhtml

integrated into the tariff setting process to guide future reforms in health system financing within LMICs.

CONCLUSION

LMICs are increasingly turning to insurance-based models of healthcare and private sector providers to increase coverage of the poor and vulnerable. To help achieve value for money within these universal health coverage goals, publicly financed insurance schemes need to account for budget constraints, encourage efficient health service delivery and use good quality evidence transparently in setting reimbursement rates. Documentation of the good practice and the challenges of generating cost evidence and creating costing systems for informing reimbursement decisions in resource poor settings are lacking. While there are accepted and widely used methods for generating cost information, countries need to build more sustainable cost systems and adopt more transparent systems and methodologies for translating costs into prices.

DECLARATIONS

Ethics approval and consent to participate:

Not applicable

Consent for publication:

Not applicable

Availability of data and materials:

Not applicable.

Contributorship Statement

LG, HAS, SG, AM collaborated on the concept and design. Data extraction, analysis and interpretation were carried out by LG and SG. The manuscript was drafted by LG, SG and HAS. Critical revisions of the paper for important intellectual content were made by LG, SG, HAS and AM. Funding was obtained by LG and AM and supervision was carried out by AM.

For peer review only - http://bmjopen.bmj.com/site/about/guidelines.xhtml

Competing Interests

HS contributed to this study whilst being employed for the Center for Global Development. HS is now an employee for GSK and holds shares in the GSK group of companies; all other authors declare no competing interests. LG declares fees for postgraduate teaching at London School of Hygiene and Tropical Medicine. AM and SG declare no competing interests.

Funding

This work was supported by the Bill & Melinda Gates Foundation Grant no: INV-003239.

Acknowledgements

Grateful thanks to Yi-Ling Chi and Lydia Regan at Center for Global Development for valuable comments and advice to the search strategy.

For peer review only

REFERENCES

1 Barber SL, Lorenzoni L, Ong P. *Price Setting and Price Regulation in Health Care*. OECD 2019. doi:10.1787/ed3c16ff-en

2 Tan SY, Melendez-Torres GJ. Do prospective payment systems (PPSs) lead to desirable providers' incentives and patients' outcomes? A systematic review of evidence from developing countries. *Health Policy Plan* 2018;**33**:137–53. doi:10.1093/HEAPOL/CZX151

3 Robyn PJ, Sauerborn R, Bärnighausen T. Provider payment in community-based health insurance schemes in developing countries: a systematic review. *Health Policy Plan* 2013;**28**:111–22. doi:10.1093/heapol/czs034

4 Langenbrunner JC, Cashin C, O'Dougherty S. *Designing and Implementing Health Care Provider Payment Systems : How-To Manuals*. 2009.

5 Government of Australia. Australian Public Hospitals Cost report. 2016.https://www.ihpa.gov.au/publications/australian-public-hospitals-cost-report-2013-2014-round-18

6 Patcharanarumol, W Panichkriangkrai, W Sommanuttaweechai, A Hanson K, Wanwong, Y Tangcharoensathien V. Strategic purchasing and health system efficiency: A comparison of two financing schemes in Thailand. *PLoS One* 2018;**13**.https://pubmed.ncbi.nlm.nih.gov/29608610/ (accessed 20 Jan 2021).

7 Raulinajtys-Grzybek M. Cost accounting models used for price-setting of health services: An international review. *Health Policy (New York)* 2014;**118**:341–53. doi:10.1016/j.healthpol.2014.07.007

For peer review only - http://bmjopen.bmj.com/site/about/guidelines.xhtml

8 Bredenkamp C, Bales S, Kahur K. Transition to Diagnosis-Related Group (DRG) Payments for Health Lessons from Case Studies. Washington DC: 2020.

9 Barber SL, Lorenzoni L, Ong P. *Price Setting and Price Regulation in Health Care*. OECD 2019. doi:10.1787/ed3c16ff-en

10 Carroll N, Lord JC. The Growing Importance of Cost Accounting for Hospitals. *J Health Care Finance* 2016;**43**:172./pmc/articles/PMC6910125/ (accessed 7 Jul 2021).

11 García-Cornejo B, Pérez-Méndez JA. Influence of cost systems on efficiency. An analysis of Spanish hospitals using public national databases. *Revista de Contabilidad-Spanish Accounting Review* 2020;**23**:249–62. doi:10.6018/rcsar.365031

12 Barber SL, Lorenzoni L, Ong P. *Price Setting and Price Regulation in Health Care*. OECD 2019. doi:10.1787/ed3c16ff-en

13 Munn Z, Peters MDJ, Stern C, *et al.* Systematic review or scoping review? Guidance for authors when choosing between a systematic or scoping review approach. *BMC Med Res Methodol* 2018;**18**:1–7. doi:10.1186/S12874-018-0611-X/TABLES/1

14 Mulligan JA, Walker D, Fox-Rushby J. Economic evaluations of non-communicable disease interventions in developing countries: A critical review of the evidence base. *Cost Effectiveness and Resource Allocation* 2006;**4**:1–10. doi:10.1186/1478-7547-4-7/TABLES/5

15 Economic evaluation of communicable disease interventions in developing countries: a critical review of the published literature - Walker - 2000 - Health Economics - Wiley Online Library. https://onlinelibrary.wiley.com/doi/10.1002/1099-1050(200012)9:8%3C681::AID-HEC545%3E3.0.CO;2-X (accessed 29 Aug 2022).

16 Stenberg K, Lauer JA, Gkountouras G, *et al.* Econometric estimation of WHO-CHOICE country-specific costs for inpatient and outpatient health service delivery. *Cost Effectiveness and Resource Allocation* 2018;**16**:11. doi:10.1186/s12962-018-0095-x

17 Barber SL, O'Dougherty S, Vinyals Torres L, *et al.* Other considerations than: how much will universal health coverage cost? *Bull World Health Organ* 2020;**98**. doi:10.2471/BLT.19.238915

18 Joint Learning Network. Costing of Health Services for Provider Payment: A Practical Manual. 2014.

19 Mathauer I, Wittenbecher F. Hospital payment systems based on diagnosis-related groups: experiences in low- and middle-income countries. *Bull World Health Organ* 2013;**91**:746-756A. doi:10.2471/BLT.12.115931

20 Bredenkamp C, Bales S, Kahur K. Transition to Diagnosis-Related Group (DRG) Payments for Health Lessons from Case Studies. 2020.

21 Dianingati RS, Riewpaiboon A. Unit cost analysis of medical service in asia: A systematic review. doi:10.29090/psa.2021.02.19.034

22 Prinja S, Chauhan AS, Rajsekhar K, *et al.* Addressing the Cost Data Gap for Universal Healthcare Coverage in India: A Call to Action. *Value Health Reg Issues* 2020;**21**:226–9. doi:10.1016/J.VHRI.2019.11.003

23 National Health Authority. Journey from HBP 1.0 to HBP 2.0. 2019.

24 KPMG. Ayushman Bharat — A big leap towards Universal Health Coverage in India. 2019.

For peer review only

25 Chatterjee S, Levin C, Laxminarayan R. Unit Cost of Medical Services at Different Hospitals in India. *PLoS One* 2013;**8**:e69728. doi:10.1371/journal.pone.0069728

26 Jassim AL. Cost Measuring of Medical Services in a Hospital using Relative Value Units (RVUs) – based Process Costing System. *undefined* 2011.

27 Prinja S, Singh MP, Rajsekar K, *et al.* Translating Research to Policy: Setting Provider Payment Rates for Strategic Purchasing under India's National Publicly Financed Health Insurance Scheme. *Appl Health Econ Health Policy* 2021;:1–18. doi:10.1007/s40258-020-00631-3

28 Ocharot L, 'Sriratanaban J', Ngamkiatphaisan. Sureerat' ', *et al.* Estimating uncompensated medical care cost as a result of adverse events in a university hospital in Thailand. *Asian Biomedicine* 2016;**10**:261–7.

29 Riewpaiboon A. Standard cost list for economic evaluation in Thailand. *Value in Health* 2012;**15**:A645–A645.http://www.embase.com/search/results?subaction=viewrecord&from=export&id=L70915622%5Cnhttp://dx.doi.org/10.1016/j.jval.2012.08.255

30 Minh H van, Phuong NK, Özaltın A, *et al.* Costing of commune health station visits for provider payment reform in Vietnam. *Glob Public Health* 2015;**10**. doi:10.1080/17441692.2014.944929

31 Vo TQ, Chaikledkaew U, Minh H, *et al.* Development of Relative Value Units for Unit Cost analysis of Medical Services in Vietnam. *Asian J Pharm* 2018;**12**:S19–26.

32 Vo TQ, Chaikledkaew U, Minh H, *et al.* Hospital cost analysis in developing countries: A methodological comparison in Vietnam. *Asian J Pharm* 2018;**12**:S8–18.

33 Ministry of Health Republic of Indonesia. THE COSTS OF DELIVERING HEALTH SERVICES IN INDONESIA: REPORT ON A PROSPECTIVE SURVEY 2010-2011. 2012.

34 Dianingati RS, Riewpaiboon A, Youngkong S. Indonesia Hospital Cost Analysis: a Micro-Costing Approach. *Jurnal Kesehatan Masyarakat* 2019;**14**. doi:10.15294/kemas.v14i3.15627

35 Doshmangir L, Rashidian A, Kouhi F, *et al.* Setting health care services tariffs in Iran: half a century quest for a window of opportunity. *Int J Equity Health* 2020;**19**. doi:10.1186/s12939-020-01224-1

36 Ghaffari S, Doran C, Wilson A, *et al.* Investigating DRG cost weights for hospitals in middle income countries. *Int J Health Plann Manage* 2009;**24**:251–64. doi:10.1002/hpm.948

37 Rasiah D. Why Activity Based Costing (ABC) is still tagging behind the traditional costing in Malaysia? *Journal of Applied Finance & Banking* 2011;:83–106.

38 Ali Jadoo SA, Aljunid SM, Nur AM, *et al.* Development of MY-DRG casemix pharmacy service weights in UKM Medical Centre in Malaysia. *DARU Journal of Pharmaceutical Sciences* 2015;**23**. doi:10.1186/s40199-014-0075-4

39 Martin A. Cambodia Hospital Costing and Financial Management Study. 2012.

40 Jacobs B, Hui K, Lo V, *et al.* Costing for universal health coverage: insight into essential economic data from three provinces in Cambodia. *Health Econ Rev* 2019;**9**:29. doi:10.1186/s13561-019-0246-6

For peer review only

For peer review only - http://bmjopen.bmj.com/site/about/guidelines.xhtml

41 Mathauer I. Setting health insurance remuneration rates of private providers in Kenya: the role of costing, challenges and implications. *Int J Health Plann Manage* 2011;**26**. doi:10.1002/hpm.1038

42 Zhao C, Wang C, Shen C, *et al.* Diagnosis-related group (DRG)-based case-mix funding system, a promising alternative for fee for service payment in China. *Biosci Trends* 2018;**12**. doi:10.5582/bst.2017.01289

43 National Health Authority. Journey from HBP1.0 to HBP2.0. New Delhi: 2019.

For peer review only

For peer review only - http://bmjopen.bmj.com/site/about/guidelines.xhtml

Figures

Figure 1: PRISMA diagram

Figure 2: Number of papers by country breakdown and types of study

Figure 3. Number of papers explaining the tariff setting scheme by country

Figure 4. Number of papers by year of publication.

Figure 5 Summary of evidence on the tariff setting process for case-based hospital payment in national health insurance schemes

For peer review only

For peer review only - http://bmjopen.bmj.com/site/about/guidelines.xhtml

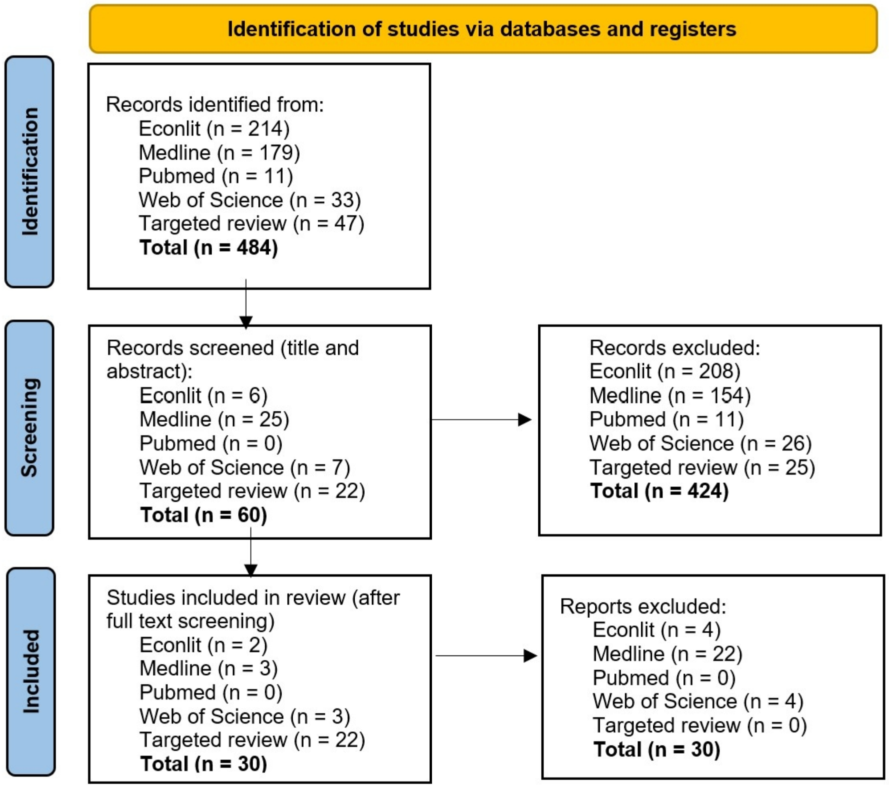

Figure 1 PRISMA diagram

109x92mm (288 x 288 DPI)

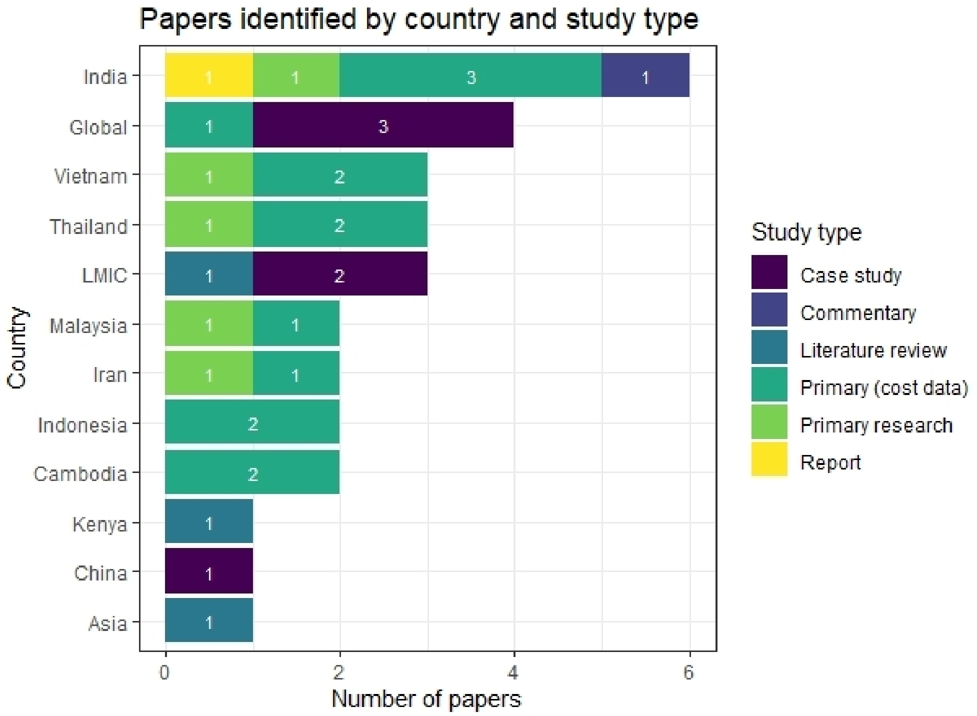

Figure 2: Number of papers by country breakdown and types of study

162x118mm (96 x 96 DPI)

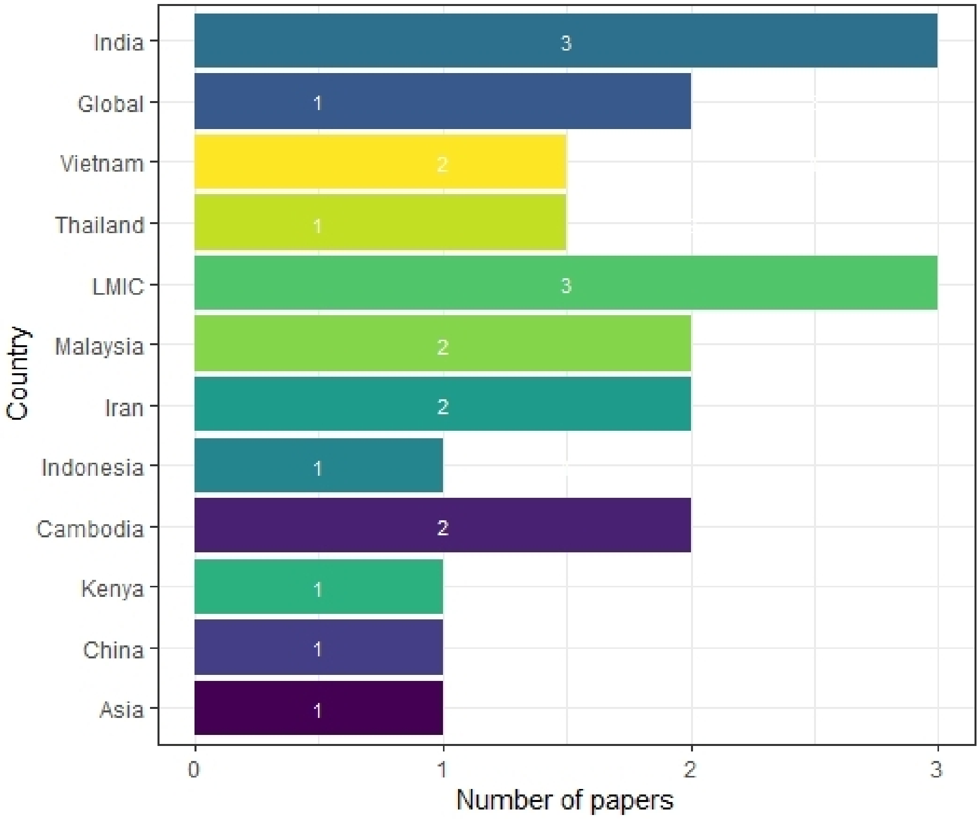

Figure 3. Number of papers explaining the tariff setting scheme by country

141x118mm (96 x 96 DPI)

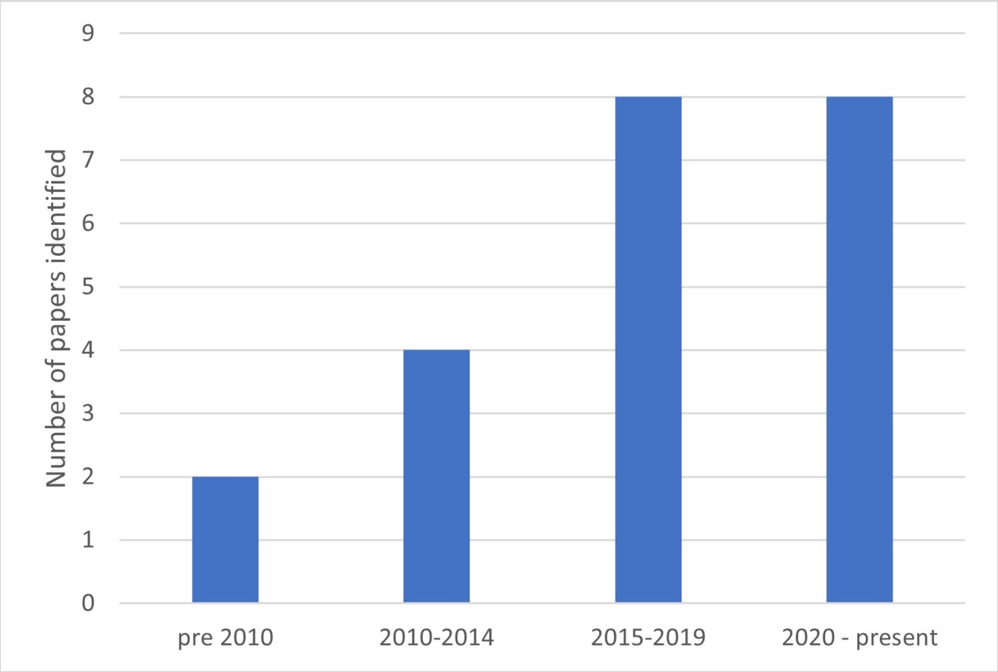

Figure 4. Number of papers by year of publication.

119x80mm (330 x 330 DPI)

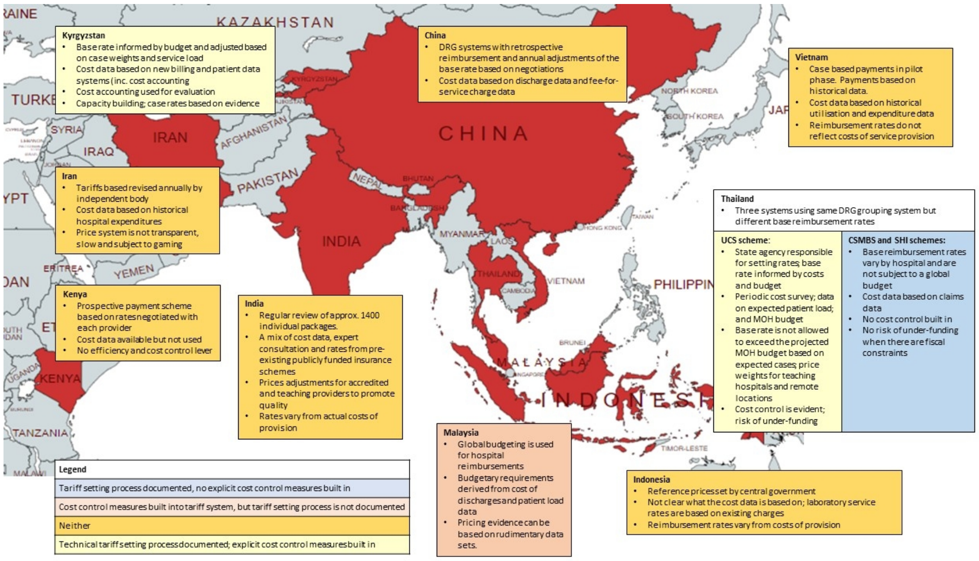

Figure 5. Summary of evidence on the tariff setting process for case-based hospital payment in national health insurance schemes

338x190mm (96 x 96 DPI)

Supplementary materials

Contents

Supplementary materials 1
Structured abstract 2
Table S1. Search strategy 4
Table S2. List of studies identified and included in the review 5
Table S3. Costing evidence for tariff setting in hospital payment schemes in LMICs 7

For peer review only

Structured abstract

Background.

Progress towards universal health coverage requires evidence based policy and price setting informed by good quality cost data systems. Establishing these systems can be complex and both resource and time intensive. and there is little documentary evidence of experiences building such systems in low-and-middle income countries (LMICs).

Objectives.

To synthesise evidence on the experiences of LMICs in institutionalizing cost data systems in order to derive lessons for the technical process of price-setting in the context of UHC goals.

Eligibility criteria.

Studies were included if they identified and/or described either the development of the national tariffs and/or the methods used to estimate or inform the tariffs for hospital services reimbursement.

Sources of evidence,

English-language publications since 2000 indexed in Pubmed, Medline, Econlit and the Web of Science.

Charting methods.

Papers were classified according to whether they explained the technical process of price-setting for reimbursements and whether they reported on the process of primary cost data collection for price-setting. We extracted information on cost data collection methods, outputs and commentary on how cost data was used as well as descriptions of the technical aspects of the tariff setting system and key strengths and challenges. A narrative review approach was used to summarise the evidence by country. Data extraction was performed by one reviewer and then checked by a second reviewer.

Results.

A total of 484 papers were initially identified of which 30 papers were considered eligible. Fourteen papers reported on primary cost data collection for price-setting purposes; 18 papers provided an

For peer review only

explanation of how cost evidence informs tariff-setting. Documented experience is largely focussed in the Asia region (n = 22) with countries at different stages of developing cost systems to inform tariff setting. Country experiences on healthcare cost accounting tend to showcase country costing experiences, methods and implementation. There is little documentation of how cost data has been incorporated into decision making and price setting. Where cost data, cost systems and costing has been used, improved transparency in decision making alongside increased service provision efficiency has followed.

Conclusions.

Countries need to build sustainable cost systems appropriate to their settings and budgets and adopt transparent processes and methodologies for translating costs into prices.

For peer review only

Table S1. Search strategy

Database	Search terms
**Web of Science**	(ALL=(("case*mix" or "cost systems" or "cost*accounting" or "ref*costs" or "resource weights" or "cost*weights" or "national reimbursement" or "DRG" or "hospital payment systems" or "fee*for*service"))) AND ALL=("LMIC" OR "low resource settings" OR "developing countries")
**Econlit**	("case*mix" or "cost systems" or "cost*accounting" or "ref*costs" or "resource weights" or "cost*weights" or "national reimbursement" or "DRG" or "hospital payment systems" or "fee*for*service").mp. [mp=heading words, abstract, title, country as subject]<br><br>No hits when combined with lmics
**Medline**	1. ("case mix" or "DRG" or "case based payment" or "hospital payment system" or "reimbursement" or "resource value unit" or "cost systems" or "cost accounting" or "reference costs" or "resource weights" or "cost weights" or "Price setting" or "service weights")<br>2. "Costs and Cost Analysis"/ or National Health Programs/ or diagnosis-related groups/ or hospitalization/<br>3. 1 OR 2<br>4. Developing Countries/<br>5. "universal health*<br>6. Universal Health Insurance/ or "Delivery of Health Care"/ or Insurance, Health/<br>7. 5 OR 6<br>8. 3 AND 4 AND 7
**PubMed**	("reference cost*" OR "reference price*") AND health* AND national* AND list*)<br>Since 2000

For peer review only

Table S2. List of studies identified and included in the review

Author	Year	Country	Aim of paper in relation to costing (rationale for inclusion)	Type of study	Cost data reported	Comments on country's cost system
Langebrunner JC et al	2009	LMIC (Kazakhstan, Kyrgyzstan)	Manual on methods for setting up provider based systems; provides methods and case study of costing accounting for case based payments	Case studies	Yes	Yes
Joint Learning Network	2014	LMIC	Costing specific resource for lmics	Case studies	Yes	Yes
Martin A	2012	Cambodia	Costing in Cambodian hospital	Primary (cost data)	Yes	Yes
Ministry of Health, Republic of Indonesia	2012	Indonesia	Report on a costing study for Indonesia	Primary (cost data)	Yes	Yes
Ghaffari S et al	2009	Iran	Costing for DRGs in Iran	Primary (cost data)	Yes	Yes
Mathauer I	2011	Kenya	Role of costing in setting insurance reimbursement rates in Kenya	Literature review	Yes	Yes
Jadoo SAA et al	2015	Malaysia	Documenting the development of DRG cost weights in pharmacy in Malaysia	Primary (cost data)	Yes	Yes
Dianingati JK et al	2019	Indonesia	Single site cost data collection to inform price setting	Primary (cost data)	Yes	Yes
Jacobs B et al	2019	Cambodia	Multiple site cost data collection to inform national policy including reimbursement rates	Primary (cost data)	Yes	Yes
Ocharot L et al	2016	Thailand	Cost implications of adverse events in DRG system	Primary (cost data)	Yes	Yes
Prinja S et al	2021	India	Comparing cost data with reimbursement rates in ABPMJAY, India	Primary (cost data)	Yes	Yes
Riewpaiboon, A. et al	2012	Thailand	Development of Relative Value Units for Unit Cost analysis of Medical Services in Thailand	Primary (cost data)	Yes	Yes
Vo TQ et al	2018a	Vietnam	Development of Relative Value Units for Unit Cost analysis of Medical Services in Vietnam	Primary (cost data)	Yes	Yes
Vo TQ et al	2018b	Vietnam	Comparison of hospital costing methods in Vietnam	Primary (cost data)	Yes	Yes
Dianingati RS et al	2021	Asia	Literature review of medical service costs in Asia	Literature review	Yes	No
Jassim AL et al	2011	India	Testing for RVU method for costing in hospitals in India	Primary (cost data)	Yes	No
Chatterjee S et al	2013	India	Hospital costing study	Primary (cost data)	Yes	No
Stenberg K et al	2018	Global	Estimating unit costs of health services at a country level based on global dataset	Primary (cost data)	Yes	-
Lian LL et al	2014	Taiwan	Assessing incentives in DRGs		No	Yes
Barber S et al	2019	Global (India, Malaysia, Thailand)	Manual and case studies on price setting for case-based payment	Case studies	No	Yes
Bredenkamp C et al	2020	Global (China, Kyrgyz Republic, Thailand)	Case studies of DRG transitions	Case studies	No	Yes

For peer review only

Author	Year	Country	Aim of paper in relation to costing (rationale for inclusion)	Type of study	Cost data reported	Comments on country's cost system
Zhao C et al	2018	China	Document China's experiences with shifting to case based payment schemes	Case studies	No	Yes
Prinja S et al	2020	India	Commentary on cost data for policy	Commentary	No	Yes
Mathauer I et al	2013	LMIC	Literature review of DRG experiences in LMICs	Literature review	No	Yes
Doshmangir L et al	2020	Iran	To document Iran's experience of tariff setting	Primary	No	Yes
Hoang VM, et al	2014	Vietnam	Reporting on costing in Vietnam for provider payment reform	Primary	No	Yes
National Health Authority	2019	India	Describes process of updating HBP package rates	Primary	No	Yes
Patcharanarumol K et al	2018	Thailand	Comparing strategic purchasing in two financing schemes	Primary	No	Yes
Rasiah, D et al	2011	Malaysia	Comparing methods for costing of health services in Malaysia	Primary	No	Yes
KPMG	2019	India	Overview of AB-PMJAY reform and financing mechanisms	Report	No	Yes
Barber S et al	2020	Global	To provide policy recommendations on estimating the cost of UHC	Case studies	No	Yes
Wagstaff A et al	2007	East Asia	Lessons learned in Asia for financing reforms including provider payment	Primary	No	Yes
Hu S et al	2008	China	Commentary	Commentary	No	No
Zeng W et al	2018	Global	Examining role of PBF in strengthening health systems	Commentary	No	No
Beck E et al	2012	LMIC	Describes the financial information required by policy makers and other stakeholders to enable them to make evidence-informed decisions and reviews the quantity and quality of the financial information available,	Literature review	No	No
Zou K et al	2020	China	Literature review of impact of case based payments in China	Literature review	No	No
Bertram M et al	2017	Global	Estimating unit costs for disease control programmes	Primary	No	No
Immunisation Costing Action Network	2018	LMIC	Costing of immunisation programmes	Primary	No	No
Jian W et al	2016	China	Assessing capacity of information system to implement DRGs (not cost system)	Primary	No	No
Watkins D et al	2020	LMIC	Resource requirement estimation of model health benefit packages (essential services)	Primary	No	No

Table S3. Costing evidence for tariff setting in hospital payment schemes in LMICs

Year	Author	Country/ Region	Type of study	Method of cost data collection	Generated output (what form does the cost data take e.g. Unit costs, DRG cost weights, base rates)	What is/was the cost data used for or what is the intended use
2021	Prinja S et al	India	Primary (cost data)	Step down allocation; mix of top down and bottom-up costing	Reference costs to inform price setting and HTA	Comparison of health benefit package reimbursement rates with costs; use in HTA
2021	Dianingati RS et al	Asia	Literature review	Methods used in hospital cost analysis in Asia include direct allocation, step-down allocation, simultaneous equation allocations, micro costing and simplified activity-based costing.	Different final outputs include average costs, patient level (micro) costs, RVUs and RCCs.	Not reported for the studies in the review
2019	Jacobs B et al	Cambodia	Primary (cost data)	Step down allocation; bottom-up costing	Costs of health services	Initial phase of establishing a routine costing system for health services
2019	Dianingati JK et al	Indonesia	Primary (cost data)	Step down allocation: micro costing	Unit cost of the laboratory services of a district hospital in Indonesia	To compare actual costs of laboratory services with government established reference prices
2018a	Vo TQ et al	Vietnam	Primary (cost data)	Step down allocation; Micro-costing	Relative value units (RVUs) for hospital services	Develop a set of RVUs for hospital services in Vietnam
2018b	Vo TQ et al	Vietnam	Primary (cost data)	1. Step down allocation; Micro-costing. 2. Hospital charges	Relative value units (RVUs) for hospital services	Comparison of different methods and identification of best method for the estimation of RVUs
2018	Stenberg K et al	Global	Primary	WHO-CHOICE service delivery unit costs based on regression analysis of country data sets	Cost per bed-day and cost per outpatient visit	Address cost information gap by producing standardised cost estimates that are comparable across countries
2016	Ocharot L et al	Thailand	Primary (cost data)	Hospital charges	Uncompensated adverse event costs for hospitalised patients	To demonstrate the cost implications of patient safety
2015	Jadoo SAA et al	Malaysia	Primary (cost data)	Step down allocation; bottom-up costing	DRG cost weights for pharmacy services	To identify the actual cost of pharmacy services by DRG case-mix group and inform reimbursement rates
2014	JLN	India Aarogyasri Hospital	Case study	Top down, direct and indirect costing for operating and capital costs.<br>For the cost of benefits packages a bottom-up approach was used. (4 hospitals; 42 procedures)	Costs of benefit packages	To set rates for 938 new benefit packages and revise estimates for pre-existing packages

For peer review only

Year	Author	Country/ Region	Type of study	Method of cost data collection	Generated output (what form does the cost data take e.g. Unit costs, DRG cost weights, base rates)	What is/was the cost data used for or what is the intended use
2014	JLN	Indonesia casemix	Case study	Top-down costing (137 hospitals)	Not specified	To estimate the cost of health services and construct cost weights for case based payments.
2014	JLN	Indonesia Health Facility	Case study	Top-down approach for both recurrent costs and capital costs. Bottom up approach to cost specific episodes of illness (200 hospitals)	Unit costs and episodes of illness	To estimate the production cost of services and drivers of cost variation among providers<br>Capitation payments
2014	JLN	Central Asian Republic	Case study	Predominantly top down, but also bottom up to obtain allocation statistics (15 hospitals)	Cost per bed-day by department	To estimate the cost per bed-day by cost centres to inform DRG weight coefficients<br><br>There was sufficient data available for top down costing which could be conducted in a relatively short period of time
2014	JLN	Malaysian DRG	Case study	Top-down approach to measure and value personnel, drugs/medical supplies, overheads, and capital resource use. Bottom up planned for ICU stays, laboratory tests and radiological interventions. (10 hospitals)		To determine the cost of delivering health services in government hospitals to inform budgetary requirements<br><br>Top down approach was used most of the time, but for items that were heterogenous in their resource use or expensive a bottom up approach was more suitable
2014	JLN	India (PHFI)	Case study	Mixed method approach. Top down for the cost of resources consumed. Bottom up for personnel. (5 hospitals)	JLN costing exercise	PHFI used a mixed-method approach because data on resource use were not always available at the department level
2014	JLN	Phillipines	Case study	Analysis of claims. All tertiary hospitals.		Cost of health services and specific disease categories
2013	Chatterjee S et al	India	Primary (cost data)	Step down allocation; bottom up costing	Cost per OP visit, cost per bed day; cost per emergency visit, cost per OT case	Informs hospital administrators, to improve efficiency and demonstrate the feasibility of hospital cost analysis in India

For peer review only

Year	Author	Country/ Region	Type of study	Method of cost data collection	Generated output (what form does the cost data take e.g. Unit costs, DRG cost weights, base rates)	What is/was the cost data used for or what is the intended use
2012	Riewpaiboon A.	Thailand	Primary (cost data)	1. Reimbursement price list; 2. Bottom up/step down allocation; 3. Patient interviews	1. RVUs; 2. inpatient base rate; 3. patient non-medical costs	To develop a list of standard unit costs of medical services for Thailand for HTA.
2012	Ministry of Health, Republic of Indonesia	Indonesia	Primary (cost data)	Step-down accounting methodology	For hospital services: cost per outpatient, cost per inpatient and cost per bed-day	To better understand the cost of delivering health services across the country and inform policy on geographic resource allocation, the development of hospital payment system and capitation formulae.
2012	Martin A	Cambodia	Primary (cost data)	Top down costing/step-down cost accounting (10 hospitals)	Average cost per discharge, per inpatient data and per outpatient visit by hospital department	To link costs with funding sources and document use of funds as well as inform the revision of the National Charter on Health Financing including provider payment reforms.
2011	Mathauer I	Kenya	Literature review	Most recent, multi-site costing studies<br>1. Bottom up, ingredients costing from (11 faith-based provider hospitals -); 2. Step down cost accounting model (22 private for profit hospitals)	Cost per case (surgical, non-surgical and outpatients); cost per bed-day (surgical and non-surgical)	Costing information to inform resetting health insurance remuneration rates
2011	Jassim AL et al	India	Primary (cost data)	Step down allocation; process costing	Cost per relative value unit in support cost centres (e.g. laboratory)	To improve costing accuracy and provide guidelines for improved costing at the hospital level
2009	Ghaffari S et al	Iran	Primary (cost data); cost modelling	Primary: Step down allocation; ABC costing<br>Cost modelling: DRG cost weights imported from Australia	Relative value units, DRG costs and cost weights using indirect costs, direct care services and costs of care	To guide costing efforts for case mix funding models and identify optimal method for costing given data constraints.
2009	Langebrunner JC et al	Kyrgyzstan	Case studies	Cost accounting: Step down cost allocation to departmental level, Adapted from US medicare cost reports (initially 1 public hospital)	Cost per bed day and cost per case	Informed case base payment rates in DRG system

Preferred Reporting Items for Systematic reviews and Meta-Analyses extension for Scoping Reviews (PRISMA-ScR) Checklist

SECTION	ITEM	PRISMA-ScR CHECKLIST ITEM	REPORTED ON PAGE #
**TITLE**			
Title	1	Identify the report as a scoping review.	Yes
**ABSTRACT**			
Structured summary	2	Provide a structured summary that includes (as applicable): background, objectives, eligibility criteria, sources of evidence, charting methods, results, and conclusions that relate to the review questions and objectives.	Supplement
**INTRODUCTION**			
Rationale	3	Describe the rationale for the review in the context of what is already known. Explain why the review questions/objectives lend themselves to a scoping review approach.	1-2
Objectives	4	Provide an explicit statement of the questions and objectives being addressed with reference to their key elements (e.g., population or participants, concepts, and context) or other relevant key elements used to conceptualize the review questions and/or objectives.	1,4-6
**METHODS**			
Protocol and registration	5	Indicate whether a review protocol exists; state if and where it can be accessed (e.g., a Web address); and if available, provide registration information, including the registration number.	Doesn't exist
Eligibility criteria	6	Specify characteristics of the sources of evidence used as eligibility criteria (e.g., years considered, language, and publication status), and provide a rationale.	6-7
Information sources*	7	Describe all information sources in the search (e.g., databases with dates of coverage and contact with authors to identify additional sources), as well as the date the most recent search was executed.	6
Search	8	Present the full electronic search strategy for at least 1 database, including any limits used, such that it could be repeated.	Supplement
Selection of sources of evidence†	9	State the process for selecting sources of evidence (i.e., screening and eligibility) included in the scoping review.	6-7
Data charting process‡	10	Describe the methods of charting data from the included sources of evidence (e.g., calibrated forms or forms that have been tested by the team before their use, and whether data charting was done independently or in duplicate) and any processes for obtaining and confirming data from investigators.	7
Data items	11	List and define all variables for which data were sought and any assumptions and simplifications made.	Qualitative themes identified – pg 7

For peer review only

SECTION	ITEM	PRISMA-ScR CHECKLIST ITEM	REPORTED ON PAGE #
Critical appraisal of individual sources of evidence§	12	If done, provide a rationale for conducting a critical appraisal of included sources of evidence; describe the methods used and how this information was used in any data synthesis (if appropriate).	Not done
Synthesis of results	13	Describe the methods of handling and summarizing the data that were charted.	7
**RESULTS**			
Selection of sources of evidence	14	Give numbers of sources of evidence screened, assessed for eligibility, and included in the review, with reasons for exclusions at each stage, ideally using a flow diagram.	Figure 1 and pg 7-8
Characteristics of sources of evidence	15	For each source of evidence, present characteristics for which data were charted and provide the citations.	Table 1 and supplement and pg 7-8
Critical appraisal within sources of evidence	16	If done, present data on critical appraisal of included sources of evidence (see item 12).	Not done
Results of individual sources of evidence	17	For each included source of evidence, present the relevant data that were charted that relate to the review questions and objectives.	Table 1 and supplement
Synthesis of results	18	Summarize and/or present the charting results as they relate to the review questions and objectives.	Table 1 and pgs 8-11
**DISCUSSION**			
Summary of evidence	19	Summarize the main results (including an overview of concepts, themes, and types of evidence available), link to the review questions and objectives, and consider the relevance to key groups.	11-13
Limitations	20	Discuss the limitations of the scoping review process.	13-14
Conclusions	21	Provide a general interpretation of the results with respect to the review questions and objectives, as well as potential implications and/or next steps.	14
**FUNDING**			
Funding	22	Describe sources of funding for the included sources of evidence, as well as sources of funding for the scoping review. Describe the role of the funders of the scoping review.	15

JBI = Joanna Briggs Institute; PRISMA-ScR = Preferred Reporting Items for Systematic reviews and Meta-Analyses extension for Scoping Reviews.

* Where *sources of evidence* (see second footnote) are compiled from, such as bibliographic databases, social media platforms, and Web sites.

† A more inclusive/heterogeneous term used to account for the different types of evidence or data sources (e.g., quantitative and/or qualitative research, expert opinion, and policy documents) that may be eligible in a scoping review as opposed to only studies. This is not to be confused with *information sources* (see first footnote).

‡ The frameworks by Arksey and O'Malley (6) and Levac and colleagues (7) and the JBI guidance (4, 5) refer to the process of data extraction in a scoping review as data charting.

§ The process of systematically examining research evidence to assess its validity, results, and relevance before using it to inform a decision. This term is used for items 12 and 19 instead of "risk of bias" (which is more applicable to systematic reviews of interventions) to include and acknowledge the various sources of evidence that may be used in a scoping review (e.g., quantitative and/or qualitative research, expert opinion, and policy document).

From: Tricco AC, Lillie E, Zarin W, O'Brien KK, Colquhoun H, Levac D, et al. PRISMA Extension for Scoping Reviews (PRISMAScR): Checklist and Explanation. Ann Intern Med. 2018;169:467–473. doi: 10.7326/M18-0850.

For peer review only - http://bmjopen.bmj.com/site/about/guidelines.xhtml
